# The PI3K subunits, P110α and P110β are potential targets for overcoming P-gp and BCRP-mediated MDR in cancer

**DOI:** 10.1186/s12943-019-1112-1

**Published:** 2020-01-17

**Authors:** Lei Zhang, Yidong Li, Qianchao Wang, Zhuo Chen, Xiaoyun Li, Zhuoxun Wu, Chaohua Hu, Dan Liao, Wei Zhang, Zhe-Sheng Chen

**Affiliations:** 1 State Key Laboratory of Structural Chemistry, Fujian Institute of Research on the Structure of Matter, Chinese Academy of Sciences, Fuzhou, 350002 China; 2 Department of Pharmaceutical Sciences, College of Pharmacy and Health Sciences, St. John’s University, Queens, NY 11439 USA; 3 College of Agriculture, Fujian Agriculture and Forestry University, Fuzhou, 350002 China; 4 College of Traditional Chinese Medicine, Jinan University, Guangzhou, 510632 Guangdong China; 5 Key Laboratory of Complementary and Alternative Medicine Experimental Animal Models of Guangxi, Guangxi University of Chinese Medicine, Nanning, 530200 China; 6 Institute of Plastic Surgery, Weifang Medical University, Weifang, China

**Keywords:** Cancer, P-glycoprotein (P-gp/ABCB1/MDR1), Breast cancer resistance protein (BCRP/ABCG2/ABCP/MXR), Multidrug resistance (MDR), Reversal of MDR, PI3K, P110α/PIK3CA, P110β/PIK3CB

## Abstract

**Background:**

PI3K/AKT is a vital signaling pathway in humans. Recently, several PI3K/AKT inhibitors were reported to have the ability to reverse cancer multidrug resistance (MDR); however, specific targets in the PI3K/AKT pathways and the mechanisms associated with MDR have not been found because many of the inhibitors have multiple targets within a large candidate protein pool. AKT activation is one presumed mechanism by which MDR develops during cancer treatment.

**Methods:**

The effects of inhibiting PI3K 110α and 110β by BAY-1082439 treatment and CRISPR/Cas9 knockout were examined to determine the possible functions of BAY-1082439 and the roles of PI3K 110α and 110β in the reversal of MDR that is mediated by the downregulation of P-gp and BCRP. Inhibition of AKT with GSK-2110183 showed that the downregulation of P-gp and BCRP is independent of generalized AKT inactivation. Immunofluorescence, immunoprecipitation, MTT, flow cytometry and JC-1 staining analyses were conducted to study the reversal of MDR that is mediated by P-gp and BCRP in cancer cells. An ATPase assay and a structural analysis were also used to analyze the potential mechanisms by which BAY-1082439 specifically targets PI3K 110α and 110β and nonspecifically influences P-gp and BCRP.

**Results:**

By inhibiting the activation of the PI3K 110α and 110β catalytic subunits through both the administration of BAY-1082439 and the CRISPR/Cas9 deletion of *Pik3ca* and *Pik3cb*, the ATP-binding cassette transporters P-gp/ABCB1 and BCRP/ABCG2 were downregulated, thereby reestablishing the drug sensitivity of human epidermoid carcinoma and non-small cell lung cancer (NSCLC) MDR cells. Inhibition of AKT did not reverse the MDR mediated by P-gp or BCRP. The ABC family proteins and AKT may play MDR-enhancing roles independently.

**Conclusions:**

The reversal of the dual functions of ABC-transporter-mediated and AKT-activation-enhanced MDR through the inhibition or knockout of PI3K 110α or 110β promises to improve current strategies based on combined drug treatments to overcome MDR challenges.

## Introduction

In human tissues, the phosphoinositide 3-kinase (PI 3-kinase, also known as PI3K) signaling pathway is a vital regulatory component involved in balancing metabolism, regulating a series of factors, and influencing cell survival and neuronal functions [1–10]. Among the four categories that constitute the PI3K family, class I PI3Ks are major members capable of activating protein kinase B (PKB/AKT), which is important for a series of pivotal functions involved in cell survival, proliferation, and differentiation and in intracellular trafficking [11]. Each class IA PI3K is composed of one P110 catalytic subunit (P110α/PIK3CA, P110β/PIK3CB or P110δ/PIK3CD) and one p85 regulatory subunit (p85α, p55α, p50α, p85β or p55γ). The P110α and P110β subunits are expressed in all cells [12]. In many cancers, *Pik3ca*, the gene encoding P110α, is often mutated, which increases kinase activity and leads to varying PTEN and EGFR expression [12, 13].

PI3Ks are vital components of the PI3K/AKT/mTOR pathways and are regulated by many factors (enhancers such as EGF, shh, IGF-1, insulin and CaM, and antagonists such as PTEN, GSK3β and HB9). They are critical to many functions related to AKT activation, such as cancer, chemoresistance, and longevity [14–18].

ABC membrane transporters can induce the release of various substrates, including antitumor drugs, from cells. In many MDR cancer cells, P-glycoprotein (P-gp, also known as ABCB1 and MDR1) and breast cancer resistance protein (BCRP, also known as ABCG2, ABCP and MXR), two vital ABC members, are overexpressed. P-gp mediates the extracellular release of colchicine, antibiotics, and a number of chemotherapy drugs, such as taxanes, anthracyclines, epipodophyllotoxins, and vinca alkaloids [19]. BCRP is critical for the efflux of methotrexate, anthracyclines, flavopiridol, tyrosine kinase inhibitors (TKIs), nucleoside analogs, etc. [19]. To date, no direct evidence for the regulation of an ABC transporter by a specific protein of the PI3K signaling pathway has been confirmed [20], mainly because most of the known inhibitors have multiple binding sites, and not all PI3K inhibitors show the ability to reverse MDR. AKT activation has only been hypothesized to influence ABC-mediated cancer MDR [21, 22]. In this study, we studied specific targets, the 110α and 110β subunits of PI3K, and the functions of these proteins with respect to the regulation of P-gp- and BCRP-mediated MDR during cancer treatment. Specifically, we used BAY-1082439, a highly selective PI3K inhibitor of the PI3K 110α and 110β isoforms and which is currently undergoing phase I clinical trials in patients with advanced cancer (ClinicalTrials.gov Identifier NCT01728311), in conjunction with *Pik3ca* or *Pik3cb* knockout in two MDR cancer cell lines, KB-C2 and H460/MX80, which overexpress the transporters P-gp and BCRP, respectively.

## Materials and methods

### Cells, plasmids and chemicals

Human epidermoid carcinoma KB-3-1 cell line was used as the parental drug-sensitive cell line. The MDR cell line, KB-C2, over-expressing P-gp, was induced from KB-3-1 with colchicine. Both cell lines were kindly provided by Dr. Shinichi Akiyama (Kagoshima University, Japan). Non-small cell lung cancer (NSCLC) NCl-H460 cell line and its resistant subline H460/MX20 were kindly provided by Drs. Susan E. Bates and Robert W. Robey (NIH, Bethesda, MD). H460/MX80 cells with enhanced drug-resistant ability were generated by inducing H460/MX20 cells with mitoxantrone at increasing concentrations up to 80 μM. KB-3-1 and its derivative cell subline KB-C2 and its knocked-out sublines without the PI3K 110α and 110β subunits were cultured with DMEM supplemented with 10% FBS and 1% penicillin/streptomycin in a humidified incubator containing 5% CO_2_ at 37 °C. H460, H460/MX20, H460/MX80 and the derivative gene deficient cells were cultured with RPMI1640 medium under the same conditions. CRISPR/Cas9 all-in-one plasmids encoding SgRNA and Cas9 for knock-out were purchased from GeneCopoeia Inc. (Rockville, MD). KB-C2 and H460/MX80 showing high MDR were used for evaluation of the intensive reversal of MDR after P110α or P110β subunit was knocked out in vitro by using the CRISPR/Cas9 technique. 

BAY-1082439, the PI3K 110α/β inhibitor, and GSK-2110183 (afuresertib), the inhibitor of AKT (AKT1, AKT2 or AKT3), were purchased from ChemieTek (Indianapolis, IN). Paclitaxel, mitoxantrone, doxorubicin and 3-(4,5-Dimethylthiazol-yl)-2,5-diphenyltetrazolium bromide (MTT), dimethyl sulfoxide (DMSO) were purchased form Sigma Chemical Co. (St. Louis, MO, USA). Mouse originated anti-P-gp and anti-BCRP, HRP ligated or fluorescent secondary rabbit or goat-anti mouse antibodies were purchased from Invitrogen, Thermo Fisher (Carlsbad, CA). Mouse-anti-PIK3CA antibodies were purchased from BD Biosciences (San Jose, CA) and anti-PIK3CB and anti-AKT Pan antibodies were purchased from R&D systems (Minneapolis, MN). Rabbit-anti-GSK3β, mouse-anti-P53, rabbit-anti-phospho-FOXO3a (Ser253) and rabbit-anti-Caspase-9 were purchased form Beyotime Institute of Biotechnology (Shanghai, China). Other reagents were purchased from VWR International (West Chester, PA, USA).

### MTT assay

MTT assay was used to determine the non-toxic concentrations of the inhibitors (generating a viability of over 80%). Exponentially growing cells were treated with trypsin and seeded into 96 well plates at 5 × 10^3^ cells/well. After 72 h of cell culture, 20 μL of MTT (5 mg/mL) was added to each well and incubated for 4 h. The medium containing MTT was removed carefully and 150 μL of DMSO was added to each well. The plates were agitated until the dark blue crystal dissolved completely. The absorbance was measured using an ELx 800 Universal Microplate Reader (Bio-Tek, Inc. Winooski, Vermont) at wavelength of 490 nm. All MTT assays were performed with three repeated treatments and independently repeated three times. The survival rate was statistically analyzed by SPSS 20.0 (SPSS, Chicago, IL, USA), and the curves were generated by Origin 9.0. The data are expressed as the mean ± standard error of the mean (SEM). Student’s t-test was used for analyzing the differences between groups. Differences were statistically significant at *P* < 0.05. IC_50_ was analyzed according to the survival rate-concentration curve.

### Evaluating the efficacy of P110α/β-specific inhibition by BAY-1082439 in reversing MDR in cancer cells

Cells were seeded into 96-well plates at a density of 5 × 10^3^ cells per well and cultured for 8 h. The cell cultures were treated with BAY-1082439 or GSK-2110183 for 1 or 2 h, followed by gradient concentrations of the anti-cancer drugs, starting from 0.001 μM as a non-toxic concentration. Decrease in the viability of MDR cells and in the IC_50_ of the anti-cancer drugs, together indicating MDR reversal efficacy, were evaluated by the MTT assay. Two specific drug substrates of P-gp and BCRP, colchicine and mitoxantrone, were used as antitumor drugs to induce high level of P-gp in KB-C2 or BCRP in H460/MX20 and H460/MX80, respectively.

### Examining P-gp and BCRP expression by Western blotting and immune-fluorescence

Parental drug-sensitive cells and drug-induced MDR cells were treated with inhibitor at the same conditions set for MTT assay. After 24 to 72 h of culture, Western blot and immune-fluorescence (IF) were conducted to determine the levels of P-gp and BCRP protein expression.

For the Western blot, the cells were lysed with SDS lysate reagents and boiled with 5× SDS-PAGE sample loading buffer. The proteins were then separated by electrophoresis, followed by regular protocols for the Western blot (refer to product instructions of the antibodies).

For IF analysis, KB-C2 and H460/MX80 cells were washed with PBS buffer, fixed with formaldehyde for 10 min at RT, followed by standard protocols for binding with primary and fluorescent antibodies provided by the manufacturers, and observed with Thorlabs (Newton, New Jersey) fluorescent microscope systems. The experiment was repeated independently for three times.

### Examining the efflux of doxorubicin by fluorescence microscopy and fluorescence quantification following BAY-1082439 treatment

Because doxorubicin is an fluorescent substrate of both P-gp and BCRP and can be pumped out from the cells by both ABC transporters, it was used at low concentration that can keep cells healthy and report whether BAY-1082439 can interact directly with these two transporters and inhibit the accumulation of doxorubicin within the cells. The parental and MDR cells were seeded into 96-well plates at a density of 5 × 10^3^ cells per well and cultured for 8 h. The cells were co-incubated with 10 μM of BAY-1082439 for 1 h. Doxorubicin (0.2 μM) was then used to supplement the cell cultures and co-incubated with the cells for 2 h. This short period of time and relatively low concentration of drug were applied to keep cells alive. After gently washing with PBS buffer thrice, the cells were lysed with 100 μL lysis buffer, quantified with BCA Protein Assay Kit and diluted with lysis buffer to constant concentration. Fluorescent intensity indicating doxorubicin accumulation within the attached cells was determined in a Synergy™ 4 Multi-Mode Microplate Reader (Bio Tek Instruments, Inc. Vermont, USA) (Ex/Em: 450/550 nm. Plate type: Corning 96 Flat Bottom black, clear bottom Polystyrene). The experiment was repeated three times, and the results were analyzed with SPSS 20.0 using Student’s t-test.

For the long-term consequence of the weakened efflux ability, cells were seeded at 3 × 10^3^ cells per well, cultured for 8 h, and treated with BAY-1082439 (10 μM) and doxorubicin (1 μM for the MDR cells and 0.1 μM for drug-sensitive cells to provide drug pressure and typical cell viability not less than 60%) for 72 h. Because of the inaccuracy of quantification of doxorubicin due to membrane injury of the dead or severely apoptotic cells, only fluorescent images showing distribution of doxorubicin were recorded as supplementary figures. This experiment was independently repeated three times to have statistical significance.

### ATPase activity assay

The ATPase activity of P-gp and BCRP in crude membranes of High-Five insect cells was measured in the presence of BAY-1082439 (0 to 40 μM) by PREDEASY ATPase Kits with modified protocols, as previously described [23, 24].

### Structural analysis with protein-ligand docking experiment

Structure information of the interactions between BAY-1082439 and target proteins was calculated on PatchDock Server based on molecular docking and simulation of stable structures of the complexes. PyMOL (version 1.8.x) was used to analyze the data and present the results that indicate the most stable structures, binding forces and positions, and residues or chemical groups.

### Analysis of cell apoptosis and necrosis by flow cytometry

The cells were seeded into a 6-well plate at 1.2 × 10^6^ cells per mL and cultured with respective anti-tumor drugs and the P110α/P110β inhibitor BAY-1082439 at non-cytotoxic concentration (10 μM) for 48 h. Untreated cells were used as a control. Cell apoptosis and necrosis were determined with an Annexin V-FITC Apoptosis Detection Kit (Beijing Solarbio Science & Technology Co., Ltd., Beijing) in a CytoFLEX flow cytometer (Beckman Coulter, Brea, CA). To slow down the growth rate of the cells set as control (MDR cells treated with drug only, or the drug-sensitive cells that were not influenced by BAY-1082439), we used 5% FBS as substitution for the 10% FBS in the media. And before the samples were tested in the flow cytometer, trypsin was used to treat the cells for better dispersity. The cells were dispersed with 2 mL of detection buffer provided with the detection Kit. The experiments were independently repeated three times. Results from a representative experiment were presented.

### CRISPR/Cas9 knockout and characterization for targeting gene deficiency

For the study of the regulatory functions of PI3K-110α or PI3K-110β on ABC transporters (P-gp and BCRP), the CRISPR/Cas9 knockout system was introduced for specific deletion of *Pik3ca* or *Pik3cb* gene. KB-C2 or H460/MX80 cells were detached with trypsin, washed with PBS and seeded to 48-well plates with serum-free DMEM or RPMI1640 media, respectively. To avoid the potential risk of un-specified chromosomal recombination during transfection mediated by viral vectors, polymer-based GenePORTER transfection reagents for gene delivery were mixed with plasmids HCP213150-CG01–1-10 and HCP213151-CG01–1-10, developed as sgRNA/Cas9 all-in-one expression clones respectively targeting PIK3CA (NM_006218.2) and PIK3CB (NM_006219.1) (GeneCopoeia Inc., Rockville, MD). This was then incubated at RT for 20 min, and added to the cell cultures. After incubation for 4 h, FBS was added to the cells at 10% of the volume. The cells were transfected again using the same method after 48 h of culture to achieve high transfection efficiency. During transfection and cell stabilization, anti-tumor drugs were not applied in order to maintain survival of the cells with *Pik3ca or Pik3cb* deletion that may attenuate the expression of ABC transporters and reverse MDR capability. Transcription and expression of target genes were determined by RT-PCR and Western blot after culturing the gene deficient cells for 1 month. Healthy and stable cell populations with deficiency of P110α or P110β were obtained by adequate culture.

To determine transcription of PI3K 110α subunits, Primers (5′) CCTCCACGACCATCAT (3′) and (5′) TGCCTACTGGTTCAAT (3′) were used for RT-PCR, and for PI3K 110β subunits, primers (5′) TGCTTCAGTTTCATAA (3′) and (5′) GAAGAAAAGGTCTGAC (3′) were used.

### Analysis of P110α and P110β to regulate P-gp and BCRP expression and drug mediated anti-cancer efficacy

In addition to the MTT assay, a JC-1 mitochondrial membrane potential analysis was performed to compare drug sensitivity of the cells before and after knock-out of the target genes *Pik3ca* and *Pik3cb* [25]. The protein expression of P-gp and BCRP in the knockout cell lines were analyzed with Western blot.

### Analysis of the ability of AKT-specific inhibitor GSK-2110183 to reverse P-gp- or BCRP-mediated MDR of cancer cells

MTT assay of the drug-sensitive and the drug-resistant cells was performed as mentioned previously, to evaluate whether GSK-2110183, an inhibitor specific for AKT, can reverse MDR of the cancer cells over-expressing P-gp and BCRP.

### Statistical calculation of protein expression levels by IF-Cytell analysis

Fluorescent immunoblotting analysis for protein levels was performed with Cytell™ Image Cytometer (GE Healthcare, Washington) based on 9–11 fields per well and at least three repeated experiments. Briefly, the cells were seeded into 96-well plates at a density of 5 × 10^3^ cells per well and cultured for required time with corresponding treatment in each experiment. Before analyzing, the cells were fixed with formaldehyde (4%), followed by a 10-min treatment with 0.1% of Triton-100 and then 6% of BSA. The cells were co-incubated with target-specific primary antibodies for 1 h at 37 °C, followed by adequate wash with PBS (pH 8.0) and a 30-min co-incubation with the Cy3 labeled secondary antibodies. After four times of wash with PBS, 0.5 μg mL^− 1^ of DAPi in PBS was applied to staining of the living cells and characterization. Average fluorescent intensity of the markers (Cy3) per cell was automatically calculated by dividing total Cy3 fluorescent intensity by total nuclei area (indicated by DAPi) in correspondence with the cell counts.

### Statistical analysis

All experiments based on statistical analysis were performed with at least three independent repeats. The data were statistically analyzed by SPSS 20.0 (SPSS, Chicago, IL, USA), expressed as the mean ± standard error of the mean (SEM). Student’s t-test was used for analyzing the differences between groups. Differences were statistically significant at *P* < 0.05.

## Results

### BAY-1082439 reversed the drug resistance mediated by overexpressed P-gp and BCRP in the MDR cancer cells

**Fig. 1:**
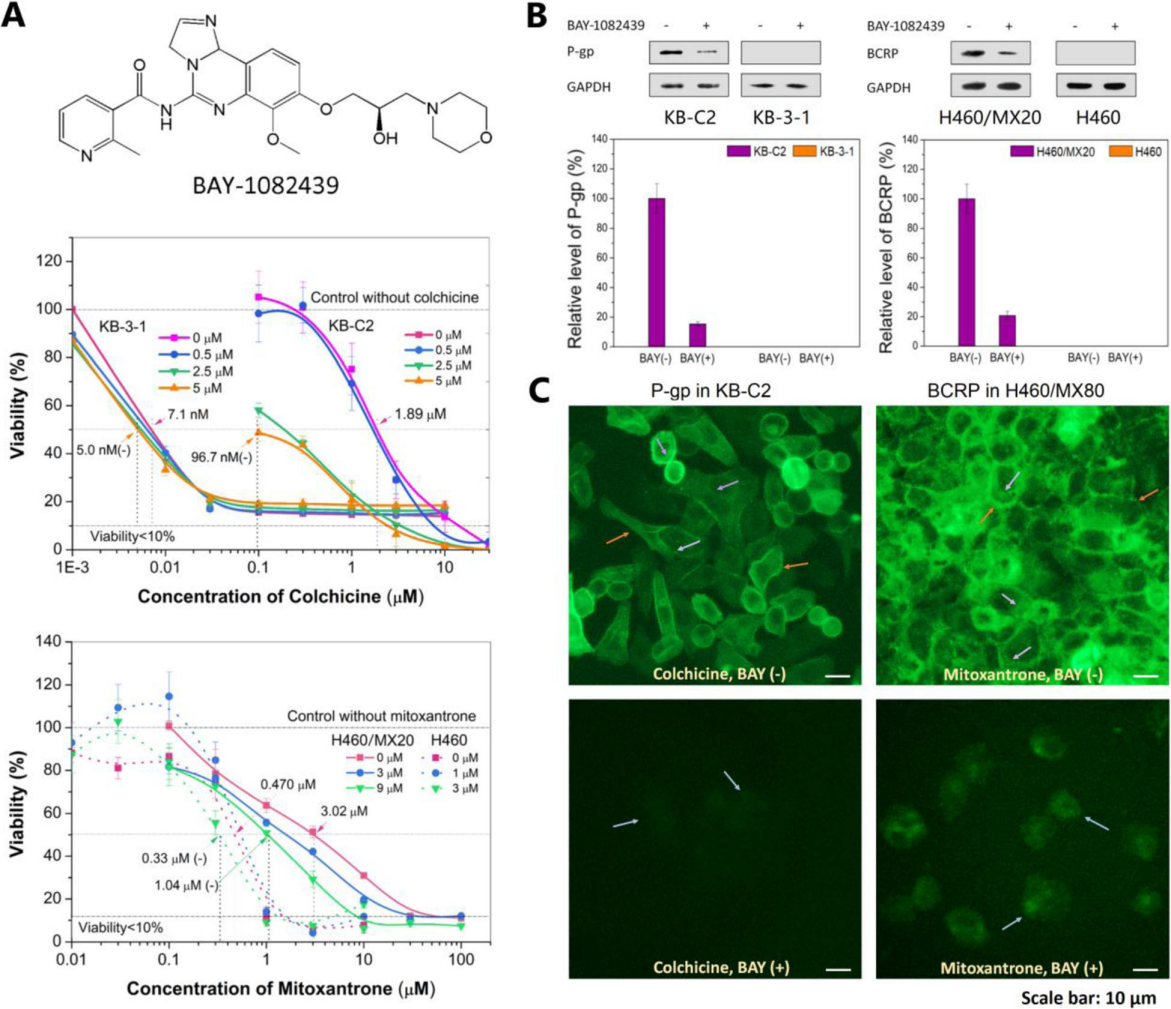
BAY-1082439 reversed MDR of cancers via down-regulating P-gp and BCRP transporters. **a** MTT assay showing the ability of BAY-1082439 to reverse MDR mediated by P-gp over-expressed in KB-C2 cells and BCRP over-expressed in H460/MX20 cells. Viability of cells without treatment with BAY-1082439 and anti-cancer drugs (colchicine for KB-C2 and mitoxantrone for H460/MX20) was set as standard control (denominator) for comparing the combined effect of BAY-1082439. The negative control, parental drug-sensitive cells KB-3-1 and H460 were treated through the same procedure. IC_50_ are indicated by arrows. **b** Down-regulation of P-gp and BCRP by BAY-1082439 targeting PI3K 110α and 110β. The cells were cultured with drugs and BAY-1082439 (BAY, 10 μM), and analyzed by Western blot. Relative quantification was carried out with ImageQuant TL based on the intensity of the bands. Colchicine at 0.7 and 0.01 μM were applied to drug-resistant KB-C2 and drug-sensitive KB-3-1 cells, respectively. Mitoxantrone at 3 and 0.3 μM were applied to drug-resistant H460/MX20 and drug-sensitive H460 cells, respectively. **c** Localization and level of P-gp and BCRP in the MDR cancer cells determined by immuno-fluorescence (IF). Positive signals on cell membrane and nuclear envelope are depicted by orange and purple arrows, respectively. Inhibition of P-gp and BCRP by BAY-1082439 is depicted by arrows in light blue. KB-C2 and H460/MX80 cells were cultured with 1 μM of colchicine and mitoxantrone, respectively, followed by co-culture with 10 μM of BAY-1082439. By 24 h and 48 h of co-culture, respectively, KB-C2 and H460/MX80 cells showing inhibition of P-gp or BCRP expression were studied by IF analysis

BAY-1082439 is a small-molecule that specifically inhibits PI3K class I P110α, P110β, and mutated forms of P110α (Fig. 1a). The survival rate of the KB-C2 and H460/MX20 MDR cells was greatly reduced in the presence of mid-to-high concentrations of the respective antitumor drugs and increasing concentrations of BAY-1082439, indicating greatly enhanced sensitivity of the cells to these anticancer drugs (Fig. 1a). The cell viability of the parental KB-3-1 and H460 cells was not apparently changed upon treatment with BAY-1082439 and the antitumor drugs. Compared to that of the parental cells, the cell viability of the KB-C2 and H460/MX20 cells was decreased, which indicated that inhibiting the PI3K 110α and 110β subunits with nontoxic or minimally toxic concentrations of BAY-1082439 can potentially reverse the P-gp- or BCRP- mediated MDR in these cells, which express the drug-efflux transporters P-gp or BCRP, a primary characteristic that distinguishes them from their parent cells.

### BAY-1082439 downregulated P-gp and BCRP expression

P-gp was downregulated in KB-C2 cells cotreated with colchicine and BAY-1082439 for only 24 h. After the dual treatment, the amount of P-gp did not exceed 18% of the P-gp in the cells treated with only colchicine (Fig. 1b). In the H460/MX20 cells, the BCRP was downregulated to approximately 20% that of the cells cotreated with mitoxantrone and BAY-1082439 after 48 h of coculture (Fig. 1b). Immunofluorescence microscopy showing the variations in the P-gp and BCRP levels and the distributions of each on the cell surface and within the cells also indicated that both P-gp and BCRP expressed by the drug-treated MDR cancer cells were downregulated by BAY-1082439 (Fig. 1c). Therefore, inhibition of PI3K 110α and/or 110β by BAY-1082439 may directly reduce the MDR protection induced by ABC transporters by downregulating their expression. These results indicated that P-gp and BCRP may have at least one common regulation factor, i.e., the P110α and/or P110β subunits in the PI3K signaling pathway.

### Protein docking analysis and ATPase assay results indicated molecular interactions between BAY-1082439 and the P-gp and BCRP transporters

**Fig. 2:**
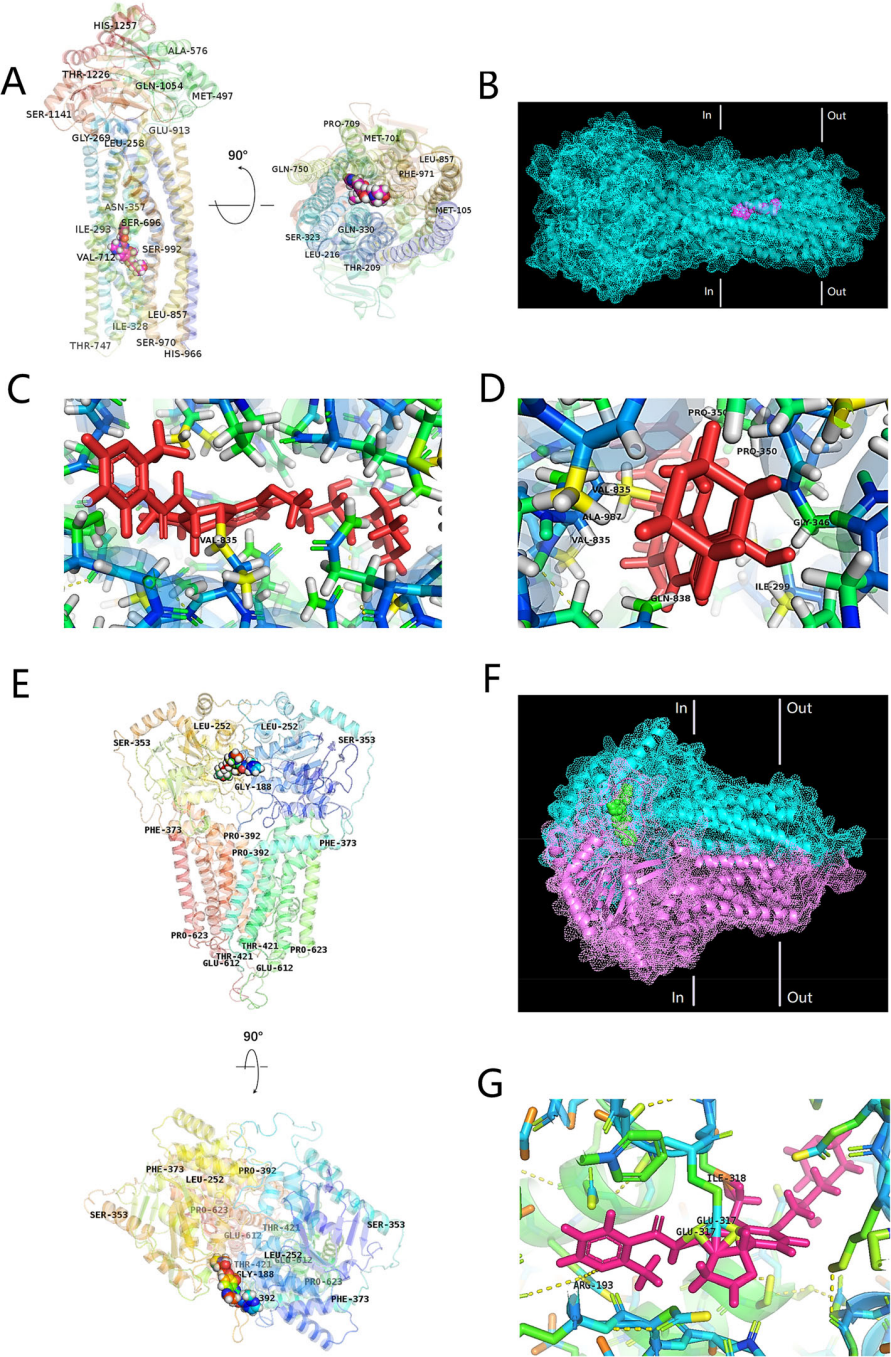
Model structure showing the interactions stabilizing BAY-1082439/P-gp and BAY-1082439/BCRP complexes. **a** Domains and positions of P-gp binding with BAY-1082439. **b** Structures showing the surface of BAY-1082439/P-gp complex. **c** Polar interaction between BAY-1082439 and P-gp. **d** Spatial structure of the cavity labeled with residues groups for P-gp to dock with BAY-1082439. **e** Binding domains and positions of BCRP docking with BAY-1082439. **f** Structures showing surface of BAY-1082439/BCRP complex. **g** Polar interaction between BAY-1082439 and BCRP

Model complex structure showed the interactions stabilizing BAY1082439/PIK3CA and BAY-1082439/PIK3CB complexes (Additional file 1: Figure S1). The binding information of BAY-1082439 with P-gp and of BAY-1082439 with BCRP was also delineated through structural analysis in a statistical prediction framework, which was used because no information has yet been reported. The results revealed interactions between BAY-1082439 and human P-gp and BCRP, as shown in Fig. 2.

P-gp comprises two symmetrical transmembrane domains (TMDs) and two cytoplasmic nucleotide-binding domains (NBDs). The NBDs form a dimer that occludes two ATPs [26]. A docking analysis generated the top 10 models showing the likely interaction between BAY-1082439 and P-gp and the localization of BAY-1082439 within P-gp. The number 1 model indicated that BAY-1082439 has the highest likelihood of binding to the central space of the TM cavity in the human P-gp that is in an outward-facing state (Fig. 2). A polar interaction was revealed between the BAY-1082439 molecule and the Val-835 residue of P-gp. These results indicated that BAY-1082439 has the ability to bind to human P-gp and may influence its ATPase activity.

The BAY-1082439/BCRP complex was predicted to have a stable structure when BAY-1082439 was bound to the internal membrane side of the BCRP dimer (Fig. 2). The BCRP dimer occluded BAY-1082439 at the near-surface cavity. In addition to surface adaptability, BAY-1082439 can dock at BCRP through the unsaturated nitrogen in the 2-pyridine interacting with Arg-193; the oxygen on the carbonyl group and the aldimine interacting with Glu-317; and -CH (adjacent to -O-C-) at 1-benzene interacting with ILE-318. Compared with a recently reported series of drug/BCRP crystal structures, this predicted binding location for BAY-1082439/BCRP was not typical. The predicted complex structure of BAY-1082439/BCRP indicated that BAY-1082439 has the ability to bind to human BCRP and may also influence its ATPase activity.

Although ATP binding to P-gp, but not ATP hydrolysis, has recently been reported to promote the release of various substrates [26], ATPase alterations can theoretically provide evidence for the induced interactions between small inhibitors/activators and ABC transporters. The results showed that the vanadate-sensitive ATPase activity of P-gp or BCRP was positively correlated with the amount of BAY-1082439 at the tested concentrations; in general, they had a fold increase of 3.3- or 1.9-fold, respectively, compared to the basal activity levels (Additional file 2: Figure S2A), indicating a stimulation of ATPase activity by BAY-1082439 in a concentration-dependent manner. The stimulation of ATPase activity may accelerate the efflux of substances under specific circumstances; however, binding of BAY-1082439 to P-gp or BCRP might also lead to P-gp or BCRP transporter mediated efflux of BAY-1082439 such that it is in competition with the antitumor drugs and results in the increase in drug accumulation within the cells. In general, our studies indicated that, upon binding to P-gp and BCRP, BAY-1082439 enhanced the ATPase activity of both P-gp and BCRP, and this finding implies the potential of BAY-1082439 to increase drug accumulation by blocking the efflux activity of both P-gp and BCRP.

### BAY-1082439 reversed drug efflux in the MDR cancer cells

As indicated by the data analyzed in Additional file 2: Figure S2B, the fluorescent intensity of KB-C2 treated with Dox (0.2 μM) was 45,407 URL mg^− 1^ protein. Co-culture with BAY-1082439 (10 μM) elevated the fluorescent intensity to 76,036 URL mg^− 1^ protein. The fluorescent intensity of KB-3-1 treated and not treated with BAY-1082439 was 211,462.7 URL mg^− 1^ and 196,077 URL mg^− 1^ protein, respectively. The fluorescent intensity of the H460/MX20 cells was elevated from 110,496.8 to 177,531.1 URL mg^− 1^ protein when the cells were treated with BAY-1082439. The fluorescent intensity of H460 with and without treatment of BAY-1082439 was 242,648.8 and 208,913.3 URL mg^− 1^ protein, respectively. The results indicated that BAY-1082439 had less inhibitory impact on the P-gp and BCRP transporters to prompt an increase of doxorubicin accumulation.

Amazingly, downregulation of P-gp and BCRP that causes long-term reversal of drug efflux behavior may have an impact on the increased drug sensitivity of KB-C2 and H460/MX80 cells in the presence of BAY-1082439, as BAY-1082439 is highly specific only for the PI3K 110α and 110β subunits (Additional file 2: Figures S2C, Additional file 3: Figure S3). Although doxorubicin is a potent intercalator of DNA, no apparent accumulation of doxorubicin appeared in the DOX (+)/BAY-1082439 (−) treatment groups even though 1 μM doxorubicin (which is a physiological concentration) was used. This outcome could have been caused by the highly efficient efflux of the doxorubicin by the KB-C2 and H460/MX80 cells with highly expressed levels of P-gp and BCRP, respectively, which endowed them with remarkably strong MDR ability. Interestingly, the florescence intensity of the sensitive cell lines was several-fold higher than that of the DOX (+)/BAY-1082439 (-)-treated MDR cells; this could be caused by the rapid increase in the accumulation of doxorubicin due to the lack of expressed P-gp or BCRP, which can efflux doxorubicin from MDR cells. According to the concentrations of doxorubicin, the drug-sensitive cells treated with 0.1 μM doxorubicin internalized lower amounts of drugs than the MDR cells treated with 1 μM doxorubicin and BAY-1082439, which inhibited P-gp and BCRP expression and reversed drug resistance.

### BAY-1082439 reversed the apoptosis resistance of the KB-C2 and H460/MX80 cells

**Fig. 3:**
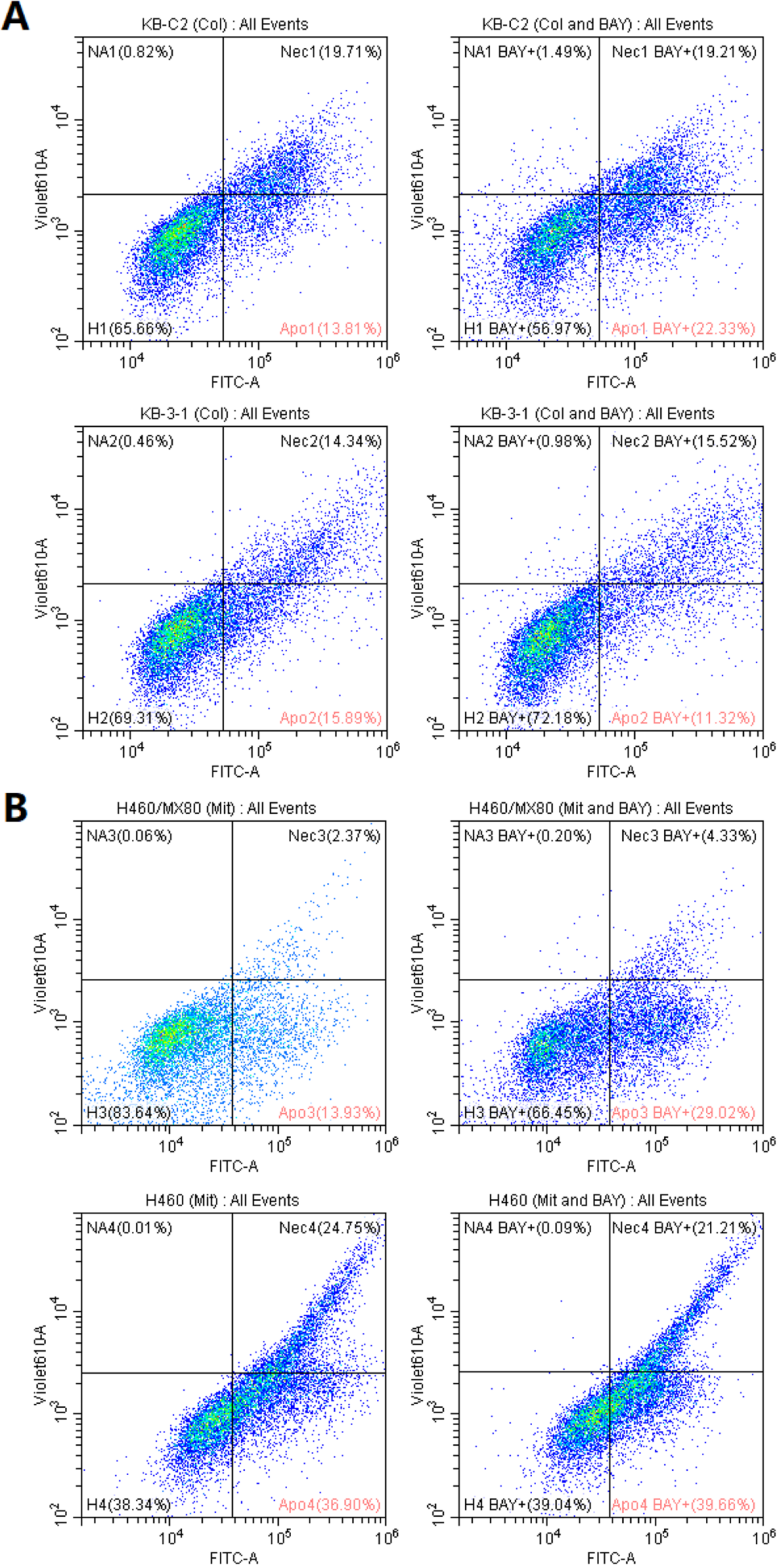
Flow cytometry analysis of BAY-1082439 in attenuating the anti-apoptosis ability of the MDR cell lines KB-C2 (**a**) and H460/MX80 (**b**) with the presence of colchicine (Col) and mitoxantrone (Mit) during 48 h of culture. The rate of cell necrosis (Nec) and apoptosis (Apo) with and without BAY-1082439 (BAY) was compared. The drug-sensitive cells KB-3-1 and H460 showing no apparent change of necrosis and apoptosis after treatment with BAY-1082439, were set as control for KB-C2 and H460/MX80, respectively. The dosage of BAY-1082439 was 10 μM. Colchicine was applied at 2 μM to KB-C2 and 0.02 μM to KB-3-1. Mitoxantrone was applied at 10 μM to H460/MX80 and 0.1 μM to H460. BAY-1082439 was applied at 10 μM to all the tested cells. Annexin V-FITC binding to phosphatidylserine (PS) that translocates to the external leaflet of the apoptotic cells was determined via FITC-A detection mode (Ex: 488 nm, Em: 525 nm). Propidium iodide (PI) that binds nucleotides of late-apoptotic or necrotic cells was determined via violet 610-A detection mode (Ex: 450 nm; Em: 610 nm) to avoid excitation of FITC. The four cell lines in healthy status were labelled with H1-H4

Both the KB-C2 and H460/MX80 cells showed a remarkable decrease in apoptosis resistance in the presence of BAY-1082439 in addition to the antitumor drugs specific to P-gp or BCRP, as indicated by flow cytometry. The ratio of apoptotic to necrotic KB-C2 cells cotreated with BAY-1082439 and colchicine was increased to approximately 42%, compared with 33% in the cells treated with colchicine only, with the major contribution made by the change in cell apoptosis, which was increased to 22% from 14% (Fig. 3a). The H460/MX80 cells treated with additional BAY-1082439 at a noncytotoxic concentration showed a two-fold rate of apoptosis compared with the control cells, of which 16% underwent apoptosis (Fig. 3b). The KB-3-1 and H460 cells did not undergo reversed antiapoptosis when treated with the same dose of BAY-1082439 in addition to the antitumor drugs.

### CRISPR/Cas9 knockout of the PI3K 110α and 110β subunits in the MDR cancer cell populations

**Fig. 4:**
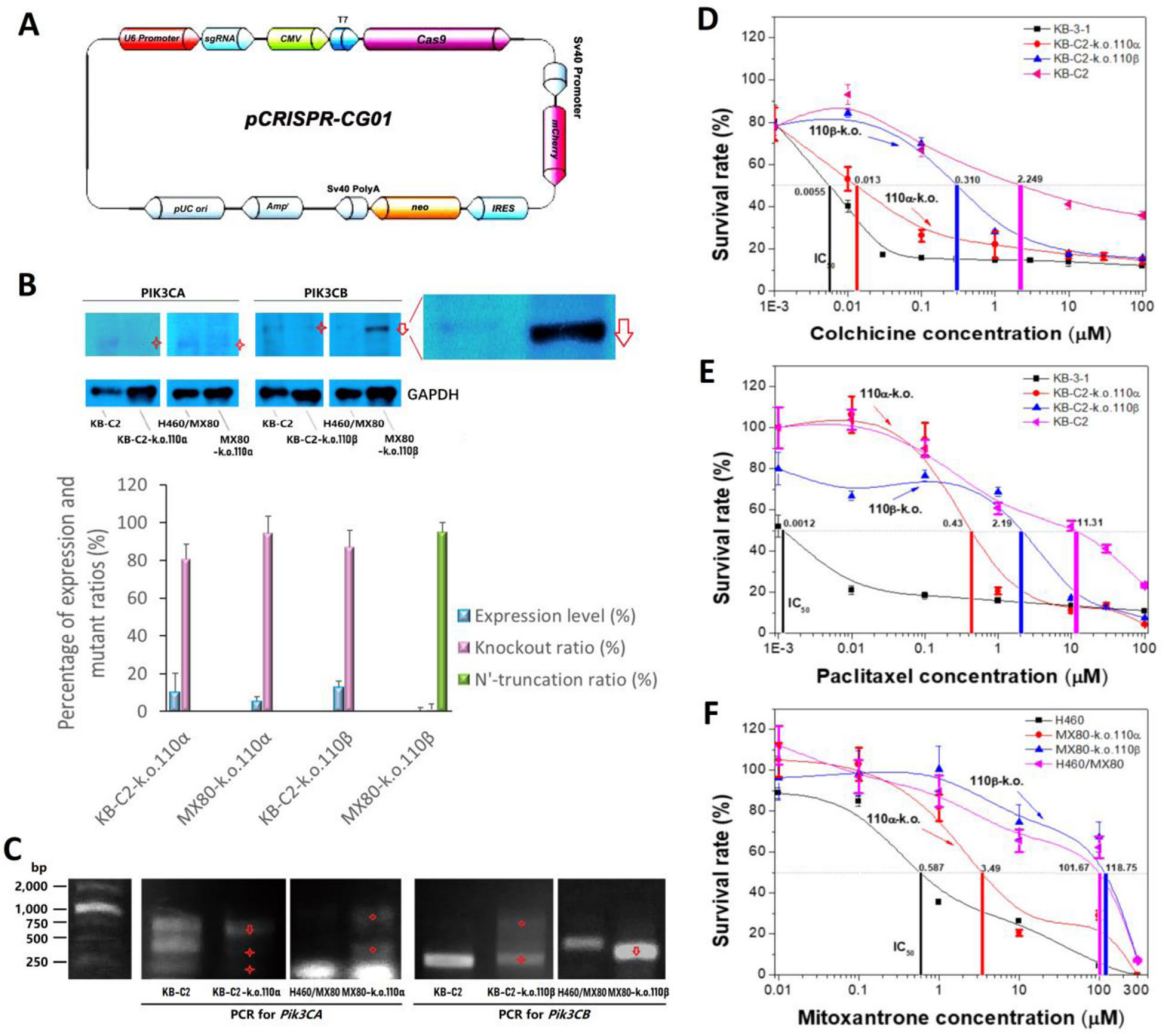
Reversal of MDR ability via knockout of target P110 subunits, including P110α (PIK3CA) and P110β (PIK3CB), from MDR cancer cell populations of KB-C2 and H460/MX80 with *pCRISPR-CG01* all-in-one plasmid. **a** Map of *pCRISPR-CG01*. **b** Analysis with Western blot confirming the knockout of PIK3CA and PIK3CB that is of low abundance in KB-C2 and H460/MX80, as compared with GAPDH. To correct possible result deviation caused by exposure saturation of partial bands, less exposure of GAPDH bands was used to indicate relative cell counts. The absence or intensity-reduction of the target bands are depicted by red stars. Truncated proteins are depicted by head-down arrows. Relative quantification was carried out with ImageQuant TL based on the intensity of the bands. Statistical calculation was made based on three independent repeats. **c** PCR as adjuvant method to characterize target P110 subunit deficiency in KB-C2 and H460/MX80 cells. Missing bands or reduced copies of the target PCR products are depicted by red stars. New bands generated by chromosomal recombination are depicted by diamonds. Truncated fragments are depicted by head-down arrows. **d**-**f** MTT assay showing changes in MDR level of the KB-C2 and H460/MX80 cells with target PI3K 110α or 110β subunits knocked out. Colchicine and paclitaxel, the substrates of P-gp, were used for evaluation of reversal of KB-C2 over-expressing P-gp. The BCRP substrate mitoxantrone was used for analysis of reversal of MDR of H460/MX80 with BCRP over-expressed. The experiments were performed at least three times

The inhibition of P110α or P110β by BAY-1082439, which in turn downregulates the P-gp and BCRP transporters, requires validation, since some PI3K inhibitors, such as CUDC-907 and afatinib, can nonspecifically inhibit other targets, such as histone deacetylase and P-gp [27, 28]. In addition, P-gp or BCRP may not be completely downregulated because of inadequate BAY-1082439 inhibition of the P110α or P110β subunit. Two cycles of gene transfection led to the successful delivery of CRISPR/Cas9 plasmids to nearly 100% of the receptor cells (Fig. 4a-c). The deletion of target genes (~ 66 kDa P110α with mutation or 110 kDa P110β in both the KB-C2 and H460/MX80 cells) in the vast majority of derivative cells and the truncation of the N′-terminus of PIK3CB in the 110β subunit-k.o. MX80 cells via gene recombination were detected at both the translational level (Fig. 4b) and the transcriptional level (Fig. 4c). Immunofluorescence also showed the knockout of target genes in most 110α subunit-k.o. and 110β subunit-k.o. KB-C2 cells and showed weak signals in the 110β subunit-k.o. H460/MX80 cells due to the N′-terminal truncation of P110β (Additional file 4: Figure S4). In comparison, nontargeted P110β in the P110α-deficient cell (110α subunit-k.o. KB-C2 or 110α subunit-k.o. H460/MX80) populations and P110α in the P110β-deficient cell (110β subunit-k.o. KB-C2 or 110β subunit-k.o. H460/MX80) populations were detected with positive fluorescence signals, indicating successful knockout of the targeted P110α or P110β subunits only. The P110α- and P110β-deficient KB-C2 cells were named KB-C2-k.o.110α and KB-C2-k.o.110β; the P110α- and P110β-deficient H460/MX80 were named MX80-k.o.110α and MX80-k.o.110β, respectively.

### Reversal of P-gp or BCRP-mediated MDR in the cells with P110α or P110 β deficiency

KB-C2 cells with either *Pik3ca* or *Pik3cb* gene deficiency were sensitive to both colchicine and paclitaxel. The IC_50_ values indicated that 110α subunit-k.o. KB-C2 cells were only 2.36-fold more sensitive to colchicine than the drug-sensitive KB-3-1 cells (Fig. 4d). Approximately 26.3% of the IC_50_ level of paclitaxel remained in the 110α subunit-k.o. KB-C2 cells compared with that of the parental cell line, KB-C2. 110β subunit-k.o. KB-C2 cells treated with colchicine and paclitaxel maintained 13.7% and 19.4% of the respective IC_50_ level in the KB-C2 cells (Fig. 4e).

The drug resistance of the 110α subunit-k.o. H460/MX80 cells was reduced to a low level, approaching that of the drug-sensitive parental H460 cells, and the IC_50_ value of the remaining 110α subunit-k.o. H460/MX80 cells was only 5–6-fold greater than that of the parental H460 cells (Fig. 4f). The MDR ability of 110β subunit-k.o. H460/MX80 cells compared with that of 110α subunit-k.o. H460/MX80 cells was relatively unchanged. Deletion of P110α in the KB-C2 cells also led to a more substantial reversal of P-gp-mediated drug resistance compared to that caused by the deletion of P110β. These results suggest that PI3K 110α could have more important function in the regulation of MDR mediated by ABC transporters, including P-gp and BCRP.

P110β deficiency in the KB-C2 and H460/MX80 cells generated differences in cell viability in the presence of respective antitumor drugs. Results from the JC-1 analysis showed severe depolarization of the mitochondrial membrane, indicating cell apoptosis of 110β subunit-k.o. KB-C2 cells after 1 μM paclitaxel treatment (Additional file 5: Figure S5). Although the MTT assay results indicated no apparent evidence for increased apoptosis of the 110β subunit-k.o. H460/MX80 cells, compared with the number of the H460/MX80 cells undergoing apoptosis, when both were treated with mitoxantrone at 10 μM, the weak green fluorescence of the JC-1 monomers indicated slightly elevated apoptosis of the 110β subunit-k.o. H460/MX80 cells.

### P-gp and BCRP but not AKT are downregulated in the P110α- or P110β-deficient cells

**Fig. 5:**
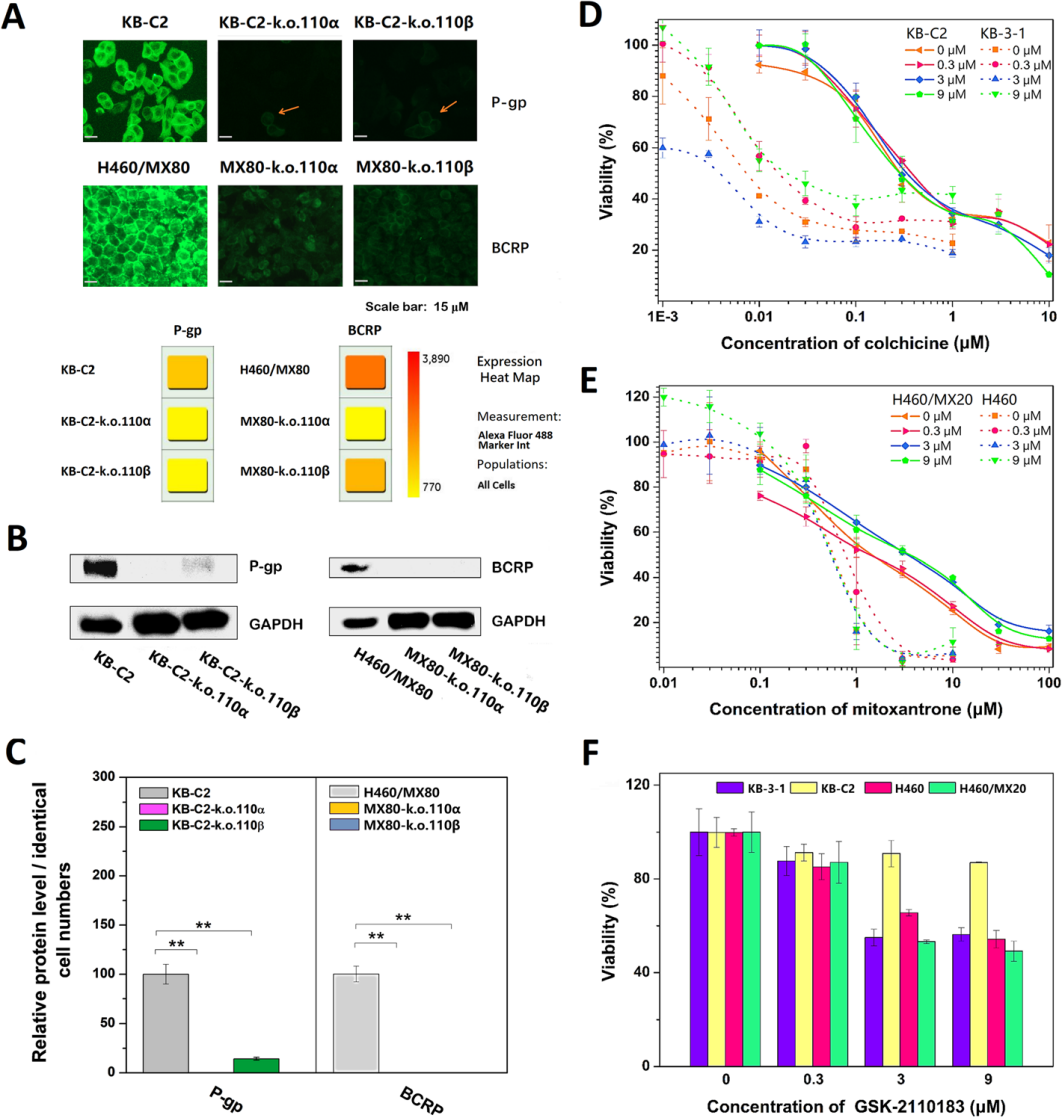
Analysis of the mechanisms for reversal of P-gp or BCRP mediated MDR ability in the MDR cancer cells with P110α or P110β knocked out. **a** Immunofluorescence microscopic characterization of down-regulation of P-gp or BCRP expression in KB-C2 and H460/MX80 cells with target P110α and P110β subunits knocked out. PI3K 110α and 110 β subunit-k.o. KB-C2 (namely KB-C2-k.o.110α and KB-C2-k.o.110β, respectively) cells expressing low level of P-gp or BCRP were depicted by arrows in orange, as most cells of these two populations were hardly be detected with positive signals, as confirmed by IF-Cytell statistical analysis according to description in the methods. The cells were seeded to 96-well plate at a density of 5 × 10^3^ cells per well. This experiment was independently repeated at least three times. **b** Western blot for the analysis of P-gp and BCRP expression in KB-C2 and H460/MX80 cells with target P110 subunits knocked out. **c** Relative protein expression level was calculated according to the band showing protein density and volume. **d** and **e** MTT assay showing poor effect of GSK-2110183 to reverse P-gp- or BCRP-mediated drug-resistance in KB-C2 or H460/MX20. **f** GSK-2110183 reduces cell viability via lower their ability to proliferate or anti-apoptosis. KB-3-1, H460, KB-C2 and H460/MX20 cells were cultured with gradient concentrations of GSK-2110183

Deficiency in P110α and P110β was associated with the downregulation of the two main MDR-relevant transporters, P-gp and BCRP, in both the KB-C2 and H460/MX80 cells, as indicated by Western blot and IF-Cytell analyses (Fig. 5a-c). As indicted by the fluorescent marker intensity value, determined by IF in combination with statistical analysis completed with a Cytell™ image cytometer, the representative expressed marker intensity was 649, 647.5, and 635.5 in the KB-C2, 110α subunit-k.o. KB-C2 and 110β subunit-k.o. KB-C2 cell populations, respectively, and was 770, 749 and 726 in the H460/MX80 and the P110α- or P110β-knockout cell populations, respectively. The expression of AKT was not altered significantly in the P110-knockout cell populations. This finding challenges a formerly presented supposition that AKT expression might be directly associated with mutated P110α subunits, a premise based on a tumor sample analysis instead of gene knockouts [29]. This phenomenon could further support the hypothesis that MDR reversal caused by P110-knockout may not be dependent on AKT expression levels.

As some researchers have previously suggested, AKT activated by PI3K might be associated with the MDR of cancer cells [30, 31]. Our studies proposed that the AKT-mediated MDR-enhancing pathway might be independent of the regulation of ABC transporter-mediated MDR and may be activated during crisis conditions, such as when the ABC transporters fail to efficiently pump toxins out of the cells.

### P-gp- or BCRP-mediated MDR effects may be independent of AKT activation

Although several studies have sought to determine whether PI3K catalytic subunits are related to ABC transporter-mediated MDR through the PI3K/AKT pathways, a direct regulatory target has not been confirmed. In this part of our study, we found contradictory results when we cotreated MDR cells with GSK-2110183 (an ATP-competitive inhibitor specific for AKT) and antitumor drugs specifically pumped out by each overexpressed ABC transporter.

None of the examined cancer cells exhibited changes in sensitivity to the antitumor drugs after being cultured with GSK-2110183; however, the cells not treated with antitumor drugs showed the inhibitory effects of GSK-2110183 treatment (Fig. 5d and e). Considering the evidence from the studies described above, we suggest that the inhibition of AKT activity does not have a regulatory effect on P-gp- or BCRP-mediated MDR in these cells.

GSK-2110183 administered alone to inhibit AKT (without antitumor drugs) inhibited the cell viability of the tested cell lines, except for the KB-C2 cells, in a dose-dependent manner (Fig. 5f). This finding may indicate that inhibition of AKT in the H460/MX80 cells generates a greater influence on cell viability than it does in the KB-C2 cells. On the basis of the combined observation that the AKT levels did not significantly change in the P110-deficient KB-C2 or H460/MX80 cells, we deemed that AKT may have a greater influence on enhancing H460/MX80 cell survival and proliferation and/or antiapoptosis, which could be independent of the MDR mediated by P-gp or BCRP.

**Fig. 6:**
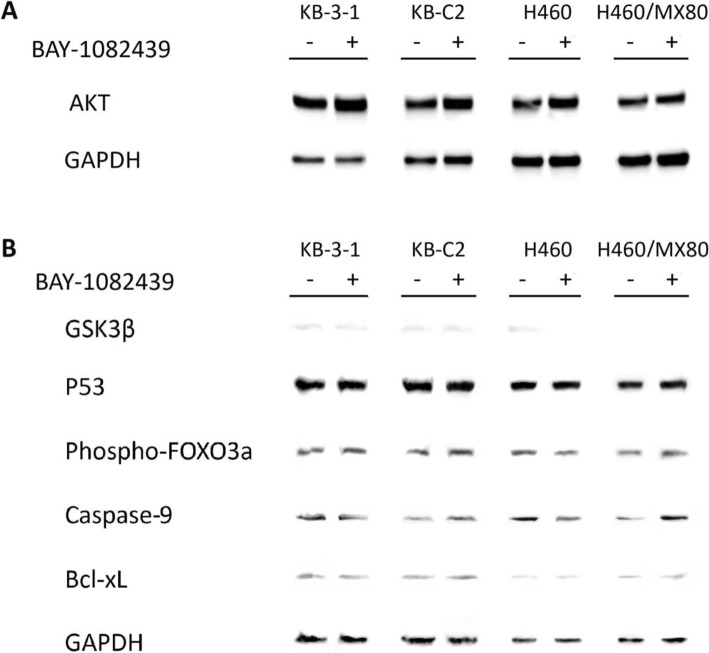
Immunoprecipitation analysis showing the effect of inhibition of P110α and P110β on the expression level of AKT and some pivotal proteins regulated by AKT. Western blot for the analysis of Pan-AKT (**a**) and series of its downstream factors (**b**) was performed after the KB-C2 and H460/MX80 MDR cells and the KB-3-1 and H460 drug sensitive parental cells were co-cultured with 10 μM of BAY-1082439 for 48 h. In addition to BAY-1082439, KB-3-1 and KB-C2 cells were also co-cultured with 0.1 and 1 μM of colchicine, and H460 and H460/MX80 cells were also co-cultured with 1 and 10 μM of mitoxantrone, respectively. Molecular for each protein: AKT: 60 kDa; GSK3β: 47 kDa; P53: 53 kDa; Phospho-FOXO3a (Ser253): 82–97 kDa; caspase-9: 46 kDa; Bcl-xL: 23.7 kDa

To demonstrate that the downregulation of P-gp or BCRP mediated by the inhibition of P110α and P110β with BAY-1082439 was independent of AKT expression, Western blot analysis was performed, and the results showed minimally enhanced expression of AKT in the cells treated with BAY-1082439 (Fig. 6). We then studied the effect of BAY-1082439 on the MDR and the parental cells on the basis of the expression levels of AKT and five pivotal proteins downstream of AKT in the PI3K/AKT pathways. In addition to Western blot analysis, a Cytell™ image cytometer was used for the quantitative analysis and comparison of the Cy3 signal intensity, which indicated the amounts of the protein targets that had bound to the antibodies (Cy3-labeled). The five other proteins are 1. glycogen synthase kinase 3 beta (GSK3β), which has multiple functions in the regulation of multiple downstream pathways (such as GYS metabolism, Myc cell cycle progression and CCND1 cell cycle progression); 2. P53 (also known as a proapoptotic protein), which regulates cell survival; 3. phosphorylated Forkhead box O3 (phospho-FOXO3a or phospho-FOXO3), which has multiple functions in the regulation of multiple factors and pathways (such as DNA-PEPCK/G6Pace metabolism, DNA-CCNDY cell cycle progression, DNA-P27KIP1/RBL2 cell cycle progression, and DNA-FasL/Bim cell survival); 4. caspase-9 (also an important apoptosis factor that regulates multiple apoptosis pathways) regulates cell survival; and 5. Bcl-xL, which is intimately related to cell survival. The results showed that, compared with the cells treated with antitumor drug alone, the MDR KB-C2 cells treated with BAY-1082439 in addition to colchicine expressed increased levels of AKT, GSK3β, phospho-FOXO3a and caspase-9 and equal levels of P53 and Bcl-xL, indicating there was no negative influence from the inhibition that P110α or P110β induced on the expression level of AKT or that of its downstream factors, indicating the ability of BAY-1082439 to induce magnified caspase-9-induced apoptosis signals and possible cell adaptation induced by other factors/pathways in addition to P110α or P110β (Fig. 6). The H460/MX80 or H460/MX20 cells treated with BAY-1082439 in addition to mitoxantrone expressed increased levels of AKT and caspase-9 and unchanged levels of the GSK3β, phospho-FOXO3a and P53 proteins, showing little negative influence from the inhibition of P110α or P110β on the level of AKT expression and that of its pivotal downstream factors, but it indicated severe apoptotic effects caused by BAY-1082439.

The drug-sensitive parental KB-3-1 cells were unaltered when the expression of AKT was changed, and all the examined factors, including caspase-9, showed little influence of BAY-1082439 on the PI3K/AKT pathways or on the growth and survival of the KB-3-1 cells (Fig. 6) Combined with the data from the BAY-1082439-induced downregulation of P-gp in the KB-C2 cells, but not in the KB-3-1 cells, the findings indicate that BAY-1082439 has a more severe effect on KB-C2 cells because of the downregulation of P-gp, which affects the efflux of drugs from the cells.

The H460 cells showed a minimal increase in AKT and P53; unchanged Bcl-xL levels; a slight decrease in GSK3β, phospho-FOXO3a and caspase-9; and unchanged levels of phospho-FOXO3a to BAY-1082439 treatment, showing good cell survivability and weak influence of BAY-1082439 on PI3K/AKT or its downstream pathways (Fig. 6). These results also explained the mechanisms inducing the increased apoptosis of the BAY-1082439-treated MDR H460/MX80 cells, but not the drug-sensitive H460 cells, as was also shown by the results from the flow cytometry analysis (Fig. 3). The major difference between the H460/MX80 and H460 cells is the degree to which downregulated BCRP in the H460/MX80 cells induced this phenomenon. Similar to the KB-C2 cells, BAY-1082439 treatment led to greater accumulation of the antitumor drugs in the cells, because of inhibited P-gp or BCRP expression, inducing increased apoptosis of these MDR cells, as suggested by caspase-9 levels and other results from the flow cytometry analysis (Fig. 3, Fig. 6). These phenomena also support the conclusion that downregulated P-gp or BCRP expression mediated by inhibition of P110α and P110β may be due to the action of pathways that are not regulated by AKT.

## Discussion

For both cancerous and normal cells, PI3K signaling pathways are pivotal to many cellular metabolic functions in a highly ordered, sophisticated system. They regulate not only metabolic processes such as protein synthesis and glycolysis/gluconeogenesis [32, 33] but also cell growth, cycle progression and cell division, proliferation, and apoptosis [34–37]. Currently, the PI3K pathway is receiving increasing attention, and far more significant functions are being revealed [38–40].

PI3K signaling pathway covers a wide and complicated network critical for the regulation of protein expression [41–43], and a series of factors belonging to different downstream functional pathways is associated with the pathway, for example, GSK3β, FOXO3A, caspase-9, and P53, which are involved in cell apoptosis and tumor suppression [44–49]; therefore, inhibition of a specific subunit is considered safer than inhibition of several nonspecific targets that operate together in PI3K-associated signaling pathways.

As shown by the results, the drug-sensitive parental cells have PI3K subunits but were not affected in terms of viability when they were treated with 10 μM BAY-1082439. This result may indicate that this concentration of BAY-1082439 might not affect the PI3K/AKT pathways or that the influence on PI3K/AKT did not cause an increase in cell death. However, P-gp and BCRP were substantially downregulated in the MDR cells, which caused the recovery of drug sensitivity, as shown by the increased cell death rate; this finding implied that BAY-1082439 reversed P-gp and BCRP expression and increased the intercellular accumulation of drugs prior to its function on PI3K/AKT/apoptosis. Therefore, the regulation of P110α and P110β on P-gp or BCRP is likely independent of the regulation of the PI3K/AKT pathways. Although this study revealed that AKT expression in the 110α- an 110β-knockout cells was unchanged, this phenomenon did not conflict with the possibly normal phosphorylation/activation of AKT by the other P110 (110β or 110α) subunit or the regulation of AKT expression level by other factors, which might be necessary for cell survival. We are carrying out studies on possible regulatory pathways that are related to downregulated P-gp or BCRP as mediated by inhibition of P110α or P110β.

**Fig. 7:**
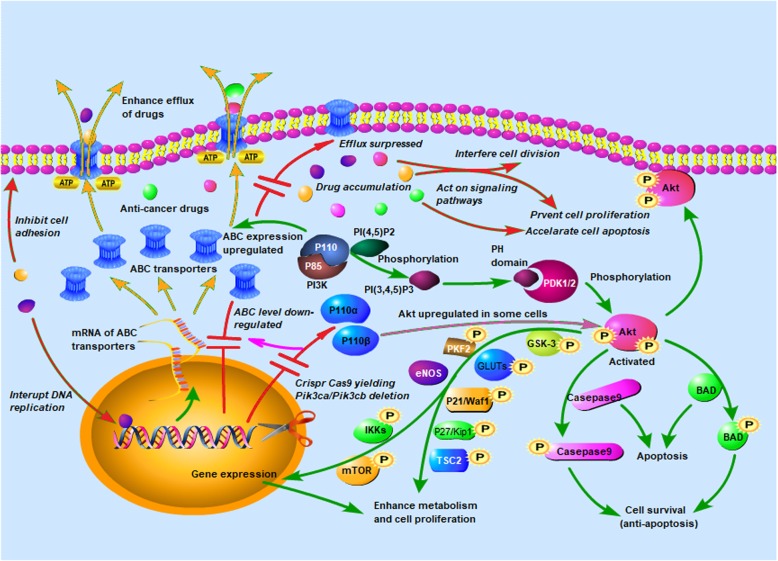
Mechanism for ABC transporters regulated by the level of PI3K 110α or 110β subunit. The level of P110 subunits may regulate P-gp and BCRP directly, altering drug-sensitivity of the cells and yielding changed cell proliferation and survival ability. Knockout of P110 subunits induced down-regulation of P-gp and BCRP, and suggested the potentiality of P110α and P110β to be applied as targets for reversal of MDR of cancers. This route can be separate from AKT activation that had been regarded as a downstream effect in PI3K/AKT signaling pathway, playing an important role in the activation of many factors through phosphorylation. Italic characters indicate the major function of CRISPR/Cas9 deletion of P110α or P110β from the MDR cancer cells

Both the KB-C2 and H460/MX20 cells cultured with the AKT inhibitor GSK did not exhibit MDR reversal. In addition to the dominant functions of P110α or P110β in regulating ABC transporters in these cells, AKT may play a separate role in enhancing drug resistance via other functions, such as inducing antiapoptosis or stimulating cell proliferation [50]. Mechanisms relevant to this dual function of P110α or P110β as specific targets for reversing cancer MDR are proposed as illustrated in Fig. 7. In this regard, signaling pathways critical for targeted regulation are under investigation in our laboratory through studies on both the protein expression network and in vivo experiments. With dual functions in the reversal of ABC-transporter-mediated and AKT-activation-enhanced MDR, inhibition or gene knockout of PI3K 110α and/or 110β is promising for the improvement to current strategies that are based on combined drug treatment to challenge MDR.

As highlighted cancer treatment strategies, most gene therapies that rely on transgene and gene deletion or silencing technologies still lack tumor specificity and are therefore limited in studies in vivo and in the clinic. Considering that PI3K signaling pathways always show particularly high activity in cancers and that PI3K subunits are frequently mutated or truncated, which may be, at least in part, a reason for the enhanced functions of the PI3K pathways during the course of tumor metagenesis and deterioration [51–54], and as Western blot analysis results showed this truncation in the KB-C2 cells but not the parental KB-3-1 cells, it is possible to design more specific knockouts based on the sequence of mutated PI3K subunits in cancer cells without interfering with the PI3K genes in normal tissues. When these types of breakthroughs are realized to resolve the current bottleneck issues related to security and specificity of gene-knockout therapeutics, a complete cure for cancer will no longer be just a vain hope.

Gene knockout based on CRISPR/Cas9 has high specificity and more advantages over target inhibition through less selective compounds and can be an ideal approach for studying a specific target. Knocking out a gene from a stable genome via CRISPR/Cas9 can be accompanied with uncertain DNA strand breaks, which may initiate a number of mechanisms involved in the self-repair in the target cells [55–57]. In addition, use of nonviral polymers as gene delivery vectors can reduce the risks of creating additional uncertain mutations. Therefore, a nonviral transgene vector-mediated CRISPR/Cas9 gene knockout in the cancer cell population can provide a safe and proper basis for evaluating the efficacy of inhibiting P110α or P110β as a target to reverse cancer MDR.

It has been reported that the ATPase activity of P-gp was increased by voruciclib at low to moderate concentrations (not exceeding 7 μM) but was inhibited by CCT129202 [58], although both compounds were categorized as inhibitors/substrates [24]. Together with a recent report indicating that ATP binding to P-gp, but not inducing hydrolysis, promotes release of the substrate [26], these findings indicate that the variance in ATPase activity showing hydrolysis by ABC transporters may be a response to a ligand binding to ABC transporters but does not necessarily indicate inhibition or activation by this ligand. Furthermore, the stimulation of ATPase activity by BAY-1082439 suggests that BAY-1082439 can bind to both P-gp and BCRP. Because of the alterations to the structure and the binding sites of the ABC proteins/molecule complexes during transportation, the ATPase and efflux activity of the transporters may also be changed.

## Conclusion

The PI3k signaling pathway is vital for many aspects of the biological functions in human and has been frequently reported with enhanced activity in cancers. Targeting a specific factor to reverse the drug-resistance without influencing the functions of normal tissue cells has become a safe and efficient strategy to overcome the MDR of cancers. In this study, we report for the first time that, through the specific inhibition of PI3K 110α and 110β subunits of PI3K with BAY-1082439 and via Crispr/Cas9 gene knockout method, two ABC transporters, P-gp/ABCB1 and BCRP/ABCG2 that contribute to the MDR of cancers were downregulated and the drug resistance was reversed in human epidermoid carcinoma and non-small cell lung cancer (NSCLC) MDR cells. Inhibition of AKT did not reverse the MDR mediated by P-gp or BCRP. Therefore, the ABC family proteins and AKT may play independent role in enhancing MDR of cancers. For the first time, P110α and P110β were demonstrated to be directly linked to MDR. The knockout of P110α and P110β is a promising strategy with high efficiency in the reduction of MDR in more than one cancer cell types and can prosperously be facilitated for administration in combination with anticancer drugs and PI3K inhibitors [59].

## Supplementary information

**Additional file 1:** **Figure S1** Model complex structure showing the interactions stabilizing BAY1082439/PIK3CA (A-C) and BAY-1082439/PIK3CB (D-F) complexes. (A) Domains and positions of BCRP binding with BAY-1082439. The region for binding to BAY-1082439 covers three functional domains of P110α, including Pro159-Tyr167 helix on the RAS binding domain (RBD), Cys257-Asp300 on the helical domain, Asn660-Ser673 helix and Met697-Leu761 helix-helix-turn structures on the C-terminal bilobal kinase domain, resulting in strong and specific inhibition on P110α catalytic subunit. (B) Polar interaction between BAY-1082439 and PIK3CA. Strong polar contact was unveiled between BAY-1082439 and Pro298 of P110α. (C) Spatial structure showing surface and residue groups for PIK3CA to dock with BAY-1082439. For the selective binding between BAY-1082439 and PI3K 110α (PIK3CA), the position for docking is possibly formed by a group of amino residues (Arg-162, Val-166, Tyr-167, Asp-258, Glu-259, Gln-296, Pro-298, ASP-300, MET-697, Tyr-698, His-701, Leu-752, Gln-760, etc.) with surface charges and cavity sizes that match BAY-1082439. (D) Domains and positions of PIK3CB binding with BAY-1082439. The site for docking of BAY-082439 and P110β involves residues Ala19-Arg114 on the adaptor-binding domain (ABD), Glu120-Ile130 helix on RBD, and Val703-Lys732 helix on N-lobe of the kinase domain. (E) Cavity structure and polar interaction between BAY-1082439 and PIK3CB. Besides shape complementarity, polar contacts exist between BAY-1082439 and Thr86, and between BAY-1082439 and Arg97. (F) Spatial structure showing surface and residue groups for PIK3CB to dock with BAY-1082439.

**Additional file 2:** **Figure S2** Activity of BAY-1082439 to alter the efflux property of P-gp and BCRP transporters in the MDR cancer cells. (A) ATPase variance of P-gp and BCRP at different concentrations of BAY-1082439. The curves corresponding to non- or low-toxic concentrations (0–10 μM) of BAY-1082439 used for MDR reversal studies was shown in magnified inset Figs. (B) Enhanced accumulation of doxorubicin within health KB-C2 and H460/MX80 cells incubated with BAY-1082439 (10 μM) and doxorubicin (0.2 μM) for less than 4 h, respectively, indicating the ability of BAY-1082439 to reverse cancer MDR. (C) BAY-1082439 induced severe cell death of MDR KB-C2 due to failure of efflux of doxorubicin during 72 h of drug treatment. The cells with especially high accumulation of DOX (red fluorescence) were detached from the plate and were distorted in cell shapes. The bright field image and fluorescent image showing the cells and DOX, respectively, were automatically merged with EVOS FL Auto Software Revision 1.7 to display distribution of DOX. DOX was used at 1 μM to provide drug pressure and typical cell viability (> 60%). BAY-1082439 was used at 10 μM.

**Additional file 3:** **Figure S3** Long-term influence of BAY-1082439 on the accumulation of doxorubicin within the MDR and drug-sensitive cell lines. (A) BAY-1082439 induced higher ratios of MDR KB-C2 and H460/MX80 cells with a weakened ability to efflux doxorubicin (DOX) during 72 h of drug treatment. The red fluorescence showed distribution and intensity of DOX accumulated within cells. The cells were seeded at 3× 10^3^ cells per well, cultured for 8 h. Then DOX was used at 1 μM to provide drug pressure for these MDR cells and support typical cell viability. BAY-1082439 for reversal of MDR was used at 10 μM prior to application of DOX. Three repeats of this experiment were performed. (B) BAY-1082439 showed no obvious function in increasing drug internalization by the parental drug-sensitive cells KB-3-1 and H460. The red fluorescence showed distribution and intensity of DOX accumulated within cells. The cells were seeded at 3× 10^3^ cells per well, cultured for 8 h. Then DOX was used at 0.1 μM to provide drug pressure for these drug-sensitive cells and keep typical cell viability. BAY-1082439 was used at 10 μM. Three repeats of this experiment were performed independently.

**Additional file 4:** **Figure S4** Immunofluorescent microscopy showing target knockout of P110 subunits. Knockout of target P110 subunit (P110α/PIK3CA or P110β/PIK3CB) in the objective cell populations showed absence of the fluorescent signals that indicate corresponding P110 (left panels), but the presence of P110 (P110α or P110β) in the positive control cells with non-target-P110 subunits (P110β or P110α) yielded in visible fluorescent signals (right panels). This determination demonstrated successful knockout of the target P110 subunit only. The cells seeded to 96-well plates (5 × 10^3^ cells per well) were cultured for 24 h, fixed with formaldehyde, pre-treated with 6% BAS in PBS, then incubated with anti-PIK3CA or anti-PIK3CB antibody for 1 h at 37 °C, followed with adequate wash with PBS and co-incubation with secondary fluorescent antibody for 30 min at 37 °C. After wash with PBS buffer three times, the samples were observed with fluorescent microscopy. The experiments were performed with 3 repeats and representative results were shown.

**Additional file 5:** **Figure S5** JC-1 analysis of the apoptosis of KB-C2 and H460/MX80 cells and the derivative cells with deficiency of P110β. JC-1 staining indicating mitochondrial membrane potential showed severe depolarization of the mitochondrial membrane as a result of severe apoptosis of 110β subunit-k.o. KB-C2 cells (namely KB-C2-k.o. 110 β), as depicted by the green arrows. KB-C2 and H460/MX80 cells without apoptosis are depicted by arrows in slight blue. Slightly apoptotic 110β subunit-k.o. H460/MX80 (namely MX80-k.o.110β) cells are depicted by arrows in pink. The KB-C2 and H460/MX80 cells were seeded into 96-well plates (5 × 10^3^ cells per well) and cultured for 8 h, followed by treatment with 1 μM of paclitaxel and 10 μM of mitoxantrone, respectively. JC-1 (2 μg mL^− 1^) was then used for cell staining for 30 min. The cells were washed with PBS and observed with fluorescent microscope. The experiments were independently repeated three times.

## Data Availability

Sequence information of PIK3CA (NM_006218.2) and PIK3CB (NM_006219.1) are available in GenBank. All other data are available in the main text or the supplementary materials. The datasets used and/or analyzed during the current study are available from the corresponding author on reasonable request.

## Abbreviations

ABD: Adaptor-binding domain
BCRP/ABCG2: Breast cancer resistance protein
CRISPR: Clustered regularly interspaced short palindromic repeats
DMSO: Dimethyl sulfoxide
DSB: Double-strand break
EGFR: Epithelial growth factor receptor
Em: Emission wavelength
Ex: Excitation wavelength
FBS: Fetal bovine serum
FITC: Fluoresceine isothiocyanate
FoXO3a: Forkhead box O3
GSK3β: Glycogen synthase kinase 3 beta
HR: Homologous recombination
IF: Immune-fluorescence
MDR: Multi-drug resistance
MMEJ: Microhomology-mediated end joining
mTOR: Mammalian Target of Rapamycin
MTT: 3-(4,5-Dimethylthiazol-yl)-2,5-diphenyltetrazolium bromide
NHEJ: Non-homologous end joining
NSCLC: Non-small cell lung cancer
P-gp/ABCB1: P-glycoprotein
PI: Propidium iodide
PI3K: Phosphoinositide 3-kinase, PI 3-kinase
PIK3CA: PI3K 110α catalytic subunit
PIK3CB: PI3K 110 β catalytic subunit
PKB/AKT: Protein kinase B
PS: Posphatidylserine
PTEN: Phosphatase and tensin homologue deleted on chromosome ten
RBD: RAS binding domain
TKI: Tyrosine kinase inhibitor
