# Supplementary material for: The PI3K subunits, P110α and P110β are potential targets for overcoming P-gp and BCRP-mediated MDR in cancer
Source: Mol Cancer. 2020 Jan 17;19:10. doi: 10.1186/s12943-019-1112-1 (PMC6966863; doi:10.1186/s12943-019-1112-1)
Supplement: Supplementary file 1 — Additional file 1: Figure S1 Model complex structure showing the interactions stabilizing BAY1082439/PIK3CA (A-C) and BAY-1082439/PIK3CB (D-F) complexes. (A) Domains and positions of BCRP binding with BAY-1082439. The region for binding to BAY-1082439 covers three functional domains of P110α, including Pro159-Tyr167 helix on the RAS binding domain (RBD), Cys257-Asp300 on the helical domain, Asn660-Ser673 helix and Met697-Leu761 helix-helix-turn structures on the C-terminal bilobal kinase domain, resulting in strong and specific inhibition on P110α catalytic subunit. (B) Polar interaction between BAY-1082439 and PIK3CA. Strong polar contact was unveiled between BAY-1082439 and Pro298 of P110α. (C) Spatial structure showing surface and residue groups for PIK3CA to dock with BAY-1082439. For the selective binding between BAY-1082439 and PI3K 110α (PIK3CA), the position for docking is possibly formed by a group of amino residues (Arg-162, Val-166, Tyr-167, Asp-258, Glu-259, Gln-296, Pro-298, ASP-300, MET-697, Tyr-698, His-701, Leu-752, Gln-760, etc.) with surface charges and cavity sizes that match BAY-1082439. (D) Domains and positions of PIK3CB binding with BAY-1082439. The site for docking of BAY-082439 and P110β involves residues Ala19-Arg114 on the adaptor-binding domain (ABD), Glu120-Ile130 helix on RBD, and Val703-Lys732 helix on N-lobe of the kinase domain. (E) Cavity structure and polar interaction between BAY-1082439 and PIK3CB. Besides shape complementarity, polar contacts exist between BAY-1082439 and Thr86, and between BAY-1082439 and Arg97. (F) Spatial structure showing surface and residue groups for PIK3CB to dock with BAY-1082439. [file 12943_2019_1112_MOESM1_ESM.docx]

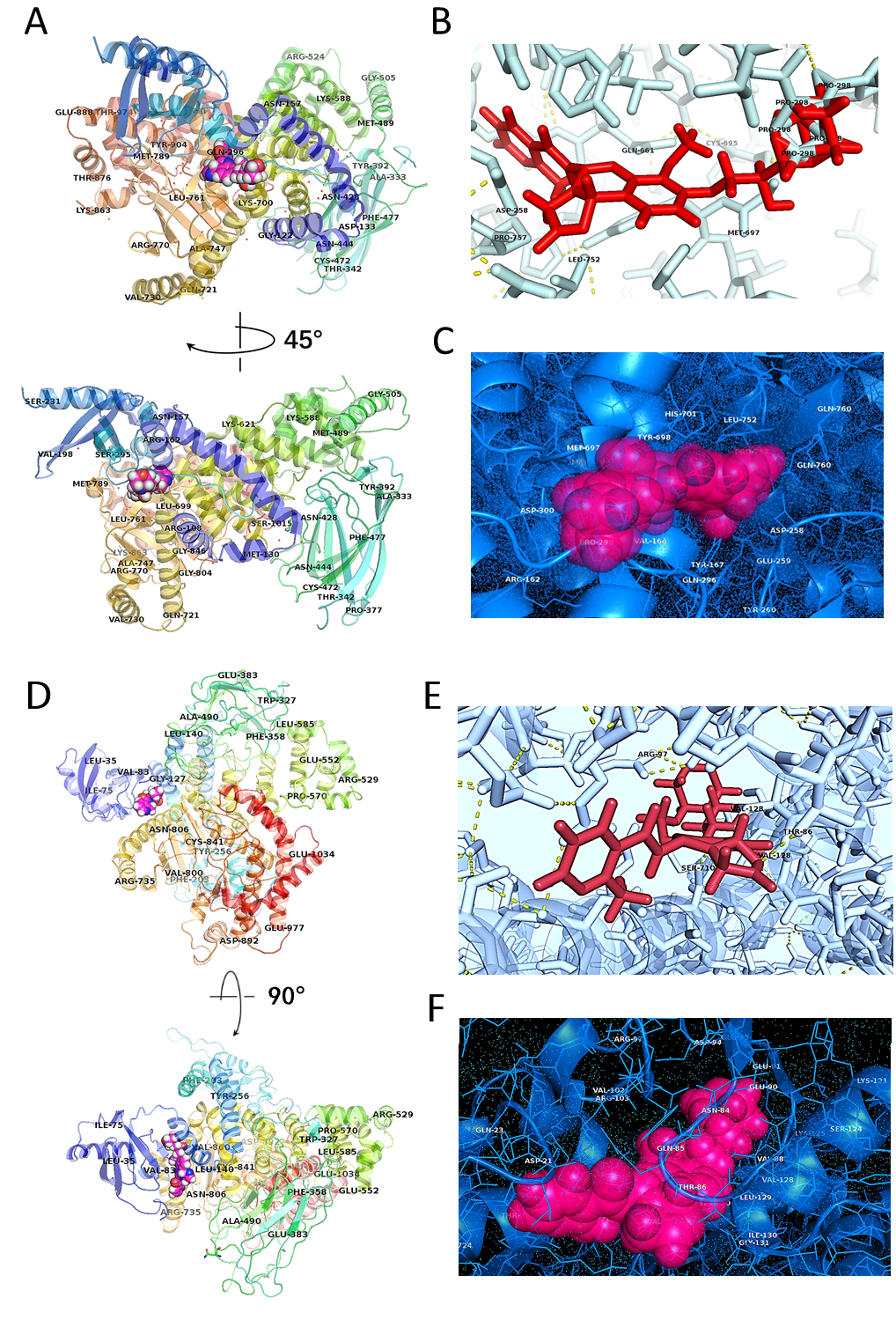


**Figure S1** **Model complex structure showing the interactions stabilizing BAY1082439/PIK3CA (A-C) and BAY-1082439/PIK3CB (D-F) complexes.** **(A)** Domains and positions of BCRP binding with BAY-1082439. The region for binding to BAY-1082439 covers three functional domains of P110α, including Pro159-Tyr167 helix on the RAS binding domain (RBD), Cys257-Asp300 on the helical domain, Asn660-Ser673 helix and Met697-Leu761 helix-helix-turn structures on the C-terminal bilobal kinase domain, resulting in strong and specific inhibition on P110α catalytic subunit. **(B)** Polar interaction between BAY-1082439 and PIK3CA. Strong polar contact was unveiled between BAY-1082439 and Pro298 of P110α. **(C)** Spatial structure showing surface and residue groups for PIK3CA to dock with BAY-1082439. For the selective binding between BAY-1082439 and PI3K 110α (PIK3CA), the position for docking is possibly formed by a group of amino residues (Arg-162, Val-166, Tyr-167, Asp-258, Glu-259, Gln-296, Pro-298, ASP-300, MET-697, Tyr-698, His-701, Leu-752, Gln-760, etc.) with surface charges and cavity sizes that match BAY-1082439. **(D)** Domains and positions of PIK3CB binding with BAY-1082439. The site for docking of BAY-082439 and P110β involves residues Ala19-Arg114 on the adaptor-binding domain (ABD), Glu120-Ile130 helix on RBD, and Val703-Lys732 helix on N-lobe of the kinase domain. **(E)** Cavity structure and polar interaction between BAY-1082439 and PIK3CB. Besides shape complementarity, polar contacts exist between BAY-1082439 and Thr86, and between BAY-1082439 and Arg97. **(F)** Spatial structure showing surface and residue groups for PIK3CB to dock with BAY-1082439.
