# Supplementary material for: The PI3K subunits, P110α and P110β are potential targets for overcoming P-gp and BCRP-mediated MDR in cancer
Source: Mol Cancer. 2020 Jan 17;19:10. doi: 10.1186/s12943-019-1112-1 (PMC6966863; doi:10.1186/s12943-019-1112-1)
Supplement: Supplementary file 2 — Additional file 2: Figure S2 Activity of BAY-1082439 to alter the efflux property of P-gp and BCRP transporters in the MDR cancer cells. (A) ATPase variance of P-gp and BCRP at different concentrations of BAY-1082439. The curves corresponding to non- or low-toxic concentrations (0–10 μM) of BAY-1082439 used for MDR reversal studies was shown in magnified inset Figs. (B) Enhanced accumulation of doxorubicin within health KB-C2 and H460/MX80 cells incubated with BAY-1082439 (10 μM) and doxorubicin (0.2 μM) for less than 4 h, respectively, indicating the ability of BAY-1082439 to reverse cancer MDR. (C) BAY-1082439 induced severe cell death of MDR KB-C2 due to failure of efflux of doxorubicin during 72 h of drug treatment. The cells with especially high accumulation of DOX (red fluorescence) were detached from the plate and were distorted in cell shapes. The bright field image and fluorescent image showing the cells and DOX, respectively, were automatically merged with EVOS FL Auto Software Revision 1.7 to display distribution of DOX. DOX was used at 1 μM to provide drug pressure and typical cell viability (> 60%). BAY-1082439 was used at 10 μM. [file 12943_2019_1112_MOESM2_ESM.docx]

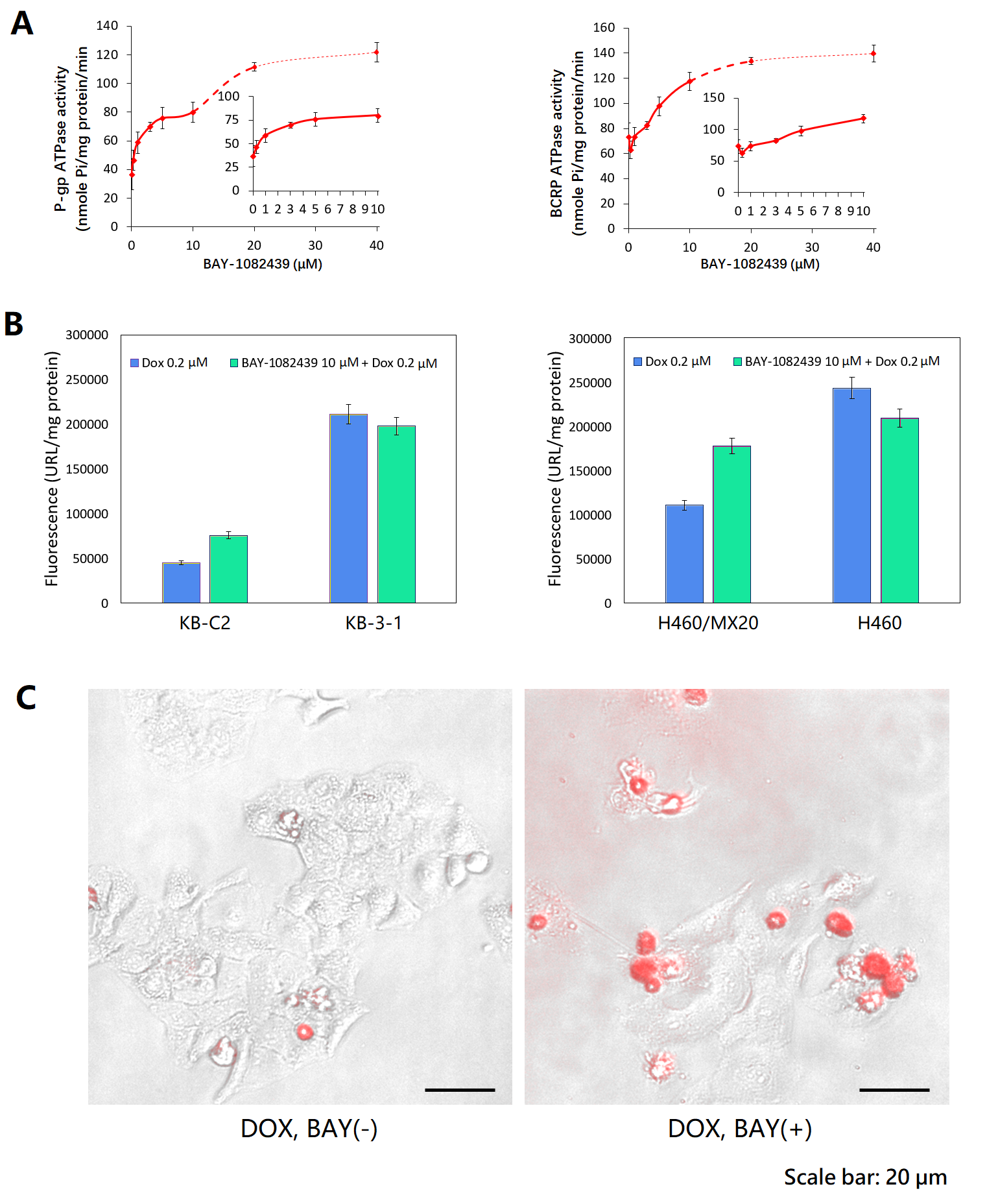


**Figure S2 Activity of BAY-1082439 to alter the efflux property of P-gp and BCRP transporters in the MDR cancer cells.** **(A)** ATPase variance of P-gp and BCRP at different concentrations of BAY-1082439. The curves corresponding to non- or low-toxic concentrations (0-10 µM) of BAY-1082439 used for MDR reversal studies was shown in magnified inset figures. **(B)** Enhanced accumulation of doxorubicin within health KB-C2 and H460/MX80 cells incubated with BAY-1082439 (10 µM) and doxorubicin (0.2 µM) for less than 4 h, respectively, indicating the ability of BAY-1082439 to reverse cancer MDR. **(C)** BAY-1082439 induced severe cell death of MDR KB-C2 due to failure of efflux of doxorubicin during 72 h of drug treatment. The cells with especially high accumulation of DOX (red fluorescence) were detached from the plate and were distorted in cell shapes. The bright field image and the fluorescent image showing the cells and DOX, respectively, were automatically merged with EVOS FL Auto Software Revision 1.7 to display distribution of DOX. DOX was used at 1 µM to provide drug pressure and typical cell viability (>60%). BAY-1082439 was used at 10 µM.
