# Supplementary material for: The PI3K subunits, P110α and P110β are potential targets for overcoming P-gp and BCRP-mediated MDR in cancer
Source: Mol Cancer. 2020 Jan 17;19:10. doi: 10.1186/s12943-019-1112-1 (PMC6966863; doi:10.1186/s12943-019-1112-1)
Supplement: Supplementary file 3 — Additional file 3: Figure S3 Long-term influence of BAY-1082439 on the accumulation of doxorubicin within the MDR and drug-sensitive cell lines. (A) BAY-1082439 induced higher ratios of MDR KB-C2 and H460/MX80 cells with a weakened ability to efflux doxorubicin (DOX) during 72 h of drug treatment. The red fluorescence showed distribution and intensity of DOX accumulated within cells. The cells were seeded at 3× 10^3^ cells per well, cultured for 8 h. Then DOX was used at 1 μM to provide drug pressure for these MDR cells and support typical cell viability. BAY-1082439 for reversal of MDR was used at 10 μM prior to application of DOX. Three repeats of this experiment were performed. (B) BAY-1082439 showed no obvious function in increasing drug internalization by the parental drug-sensitive cells KB-3-1 and H460. The red fluorescence showed distribution and intensity of DOX accumulated within cells. The cells were seeded at 3× 10^3^ cells per well, cultured for 8 h. Then DOX was used at 0.1 μM to provide drug pressure for these drug-sensitive cells and keep typical cell viability. BAY-1082439 was used at 10 μM. Three repeats of this experiment were performed independently. [file 12943_2019_1112_MOESM3_ESM.docx]

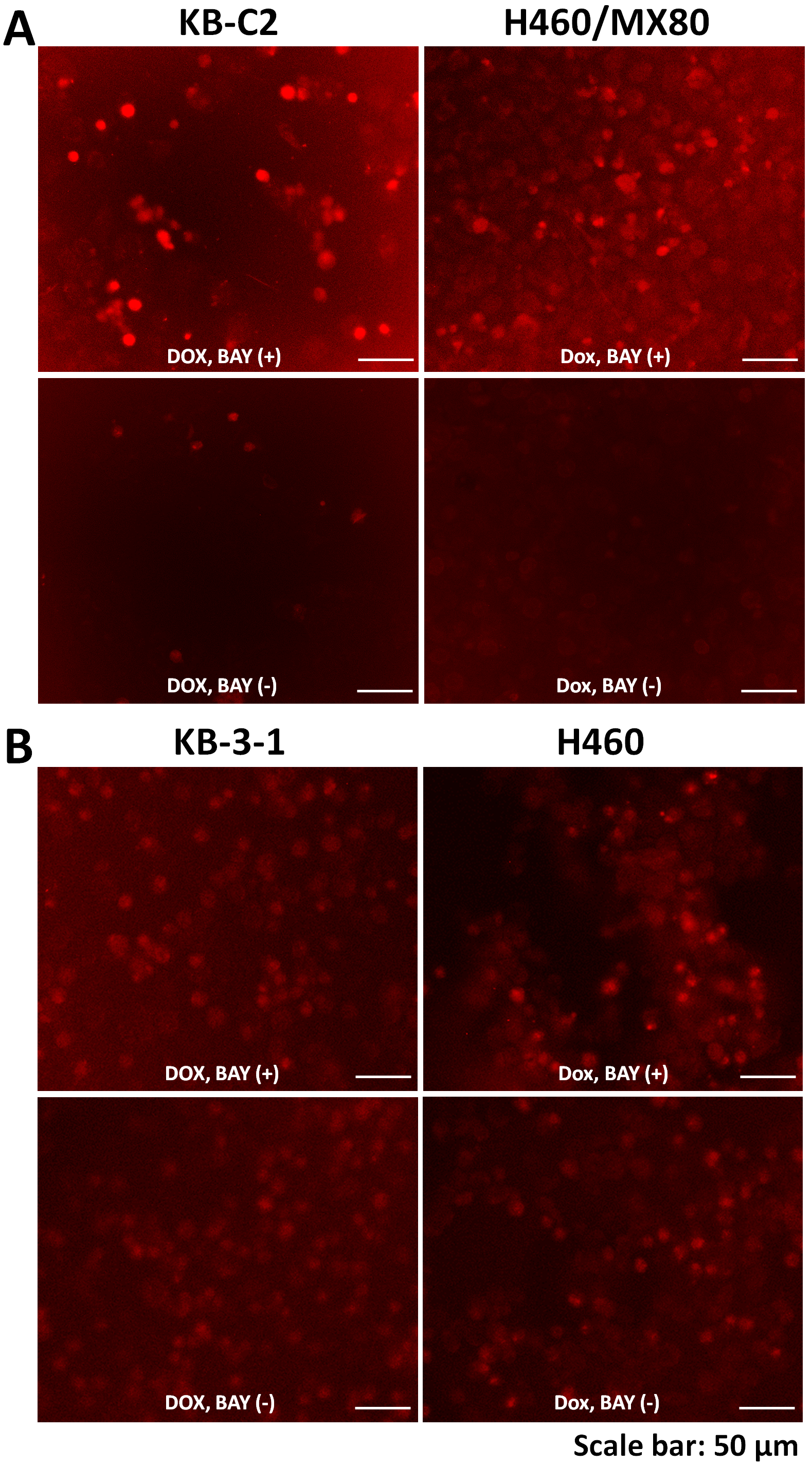


**Figure S3** **Long-term influence of BAY-1082439 on the accumulation of doxorubicin within the MDR and drug-sensitive cell lines.** **(A)** BAY-1082439 induced higher ratios of MDR KB-C2 and H460/MX80 cells with a weakened ability to efflux doxorubicin (DOX) during 72 h of drug treatment. The red fluorescence showed distribution and intensity of DOX accumulated within cells. The cells were seeded at 3× 10^3^ cells per well, cultured for 8 h. Then DOX was used at 1 µM to provide drug pressure for these MDR cells and support typical cell viability. BAY-1082439 for reversal of MDR was used at 10 µM prior to application of DOX. Three repeats of this experiment were performed. **(B)** BAY-1082439 showed no obvious function in increasing drug internalization by the parental drug-sensitive cells KB-3-1 and H460. The red fluorescence showed distribution and intensity of DOX accumulated within cells. The cells were seeded at 3× 10^3^ cells per well, cultured for 8 h. Then DOX was used at 0.1 µM to provide drug pressure for these drug-sensitive cells and keep typical cell viability. BAY-1082439 was used at 10 µM. Three repeats of this experiment were performed independently.
