# Supplementary material for: The PI3K subunits, P110α and P110β are potential targets for overcoming P-gp and BCRP-mediated MDR in cancer
Source: Mol Cancer. 2020 Jan 17;19:10. doi: 10.1186/s12943-019-1112-1 (PMC6966863; doi:10.1186/s12943-019-1112-1)
Supplement: Supplementary file 4 — Additional file 4: Figure S4 Immunofluorescent microscopy showing target knockout of P110 subunits. Knockout of target P110 subunit (P110α/PIK3CA or P110β/PIK3CB) in the objective cell populations showed absence of the fluorescent signals that indicate corresponding P110 (left panels), but the presence of P110 (P110α or P110β) in the positive control cells with non-target-P110 subunits (P110β or P110α) yielded in visible fluorescent signals (right panels). This determination demonstrated successful knockout of the target P110 subunit only. The cells seeded to 96-well plates (5 × 10^3^ cells per well) were cultured for 24 h, fixed with formaldehyde, pre-treated with 6% BAS in PBS, then incubated with anti-PIK3CA or anti-PIK3CB antibody for 1 h at 37 °C, followed with adequate wash with PBS and co-incubation with secondary fluorescent antibody for 30 min at 37 °C. After wash with PBS buffer three times, the samples were observed with fluorescent microscopy. The experiments were performed with 3 repeats and representative results were shown. [file 12943_2019_1112_MOESM4_ESM.docx]

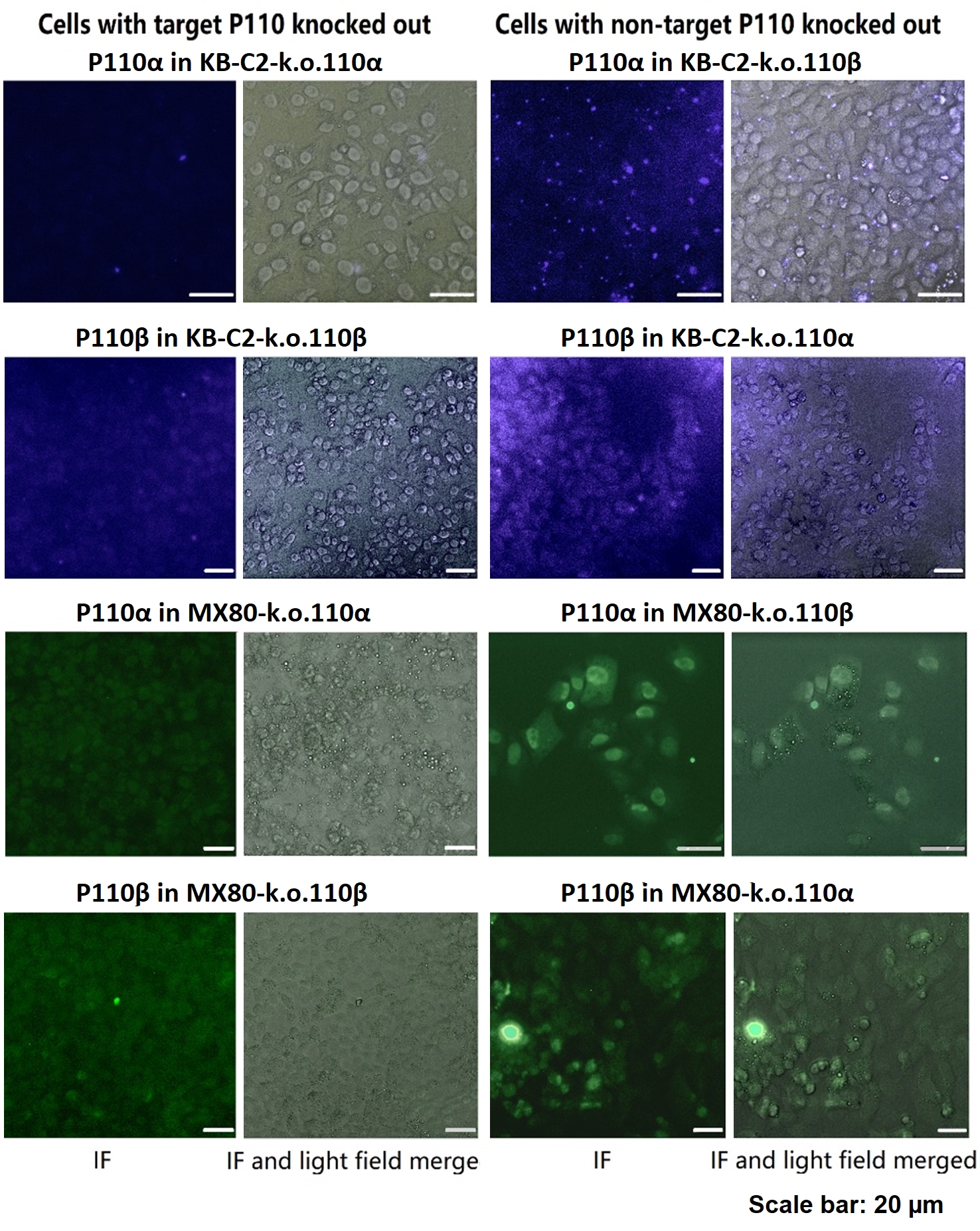


**Figure S4 Immunofluorescent microscopy showing target knockout of P110 subunits.** Knockout of target P110 subunit (P110α/PIK3CA or P110β/PIK3CB) in the objective cell populations showed absence of the fluorescent signals that indicate corresponding P110 (left panels), but the presence of P110 (P110α or P110β) in the positive control cells with non-target-P110 subunits (P110β or P110α) yielded in visible fluorescent signals (right panels). This determination demonstrated successful knockout of the target P110 subunit only. The cells seeded to 96-well plates (5×10^3^ cells per well) were cultured for 24 h, fixed with formaldehyde, pre-treated with 6% BAS in PBS, then incubated with anti-PIK3CA or anti-PIK3CB antibody for 1 h at 37 ̊C, followed with adequate wash with PBS and co-incubation with secondary fluorescent antibody for 30 min at 37 ̊C. After wash with PBS buffer three times, the samples were observed with fluorescent microscopy. The experiments were performed with 3 repeats and representative results were shown.
