# Supplementary material for: The PI3K subunits, P110α and P110β are potential targets for overcoming P-gp and BCRP-mediated MDR in cancer
Source: Mol Cancer. 2020 Jan 17;19:10. doi: 10.1186/s12943-019-1112-1 (PMC6966863; doi:10.1186/s12943-019-1112-1)
Supplement: Supplementary file 5 — Additional file 5: Figure S5 JC-1 analysis of the apoptosis of KB-C2 and H460/MX80 cells and the derivative cells with deficiency of P110β. JC-1 staining indicating mitochondrial membrane potential showed severe depolarization of the mitochondrial membrane as a result of severe apoptosis of 110β subunit-k.o. KB-C2 cells (namely KB-C2-k.o. 110 β), as depicted by the green arrows. KB-C2 and H460/MX80 cells without apoptosis are depicted by arrows in slight blue. Slightly apoptotic 110β subunit-k.o. H460/MX80 (namely MX80-k.o.110β) cells are depicted by arrows in pink. The KB-C2 and H460/MX80 cells were seeded into 96-well plates (5 × 10^3^ cells per well) and cultured for 8 h, followed by treatment with 1 μM of paclitaxel and 10 μM of mitoxantrone, respectively. JC-1 (2 μg mL− 1) was then used for cell staining for 30 min. The cells were washed with PBS and observed with fluorescent microscope. The experiments were independently repeated three times. [file 12943_2019_1112_MOESM5_ESM.docx]

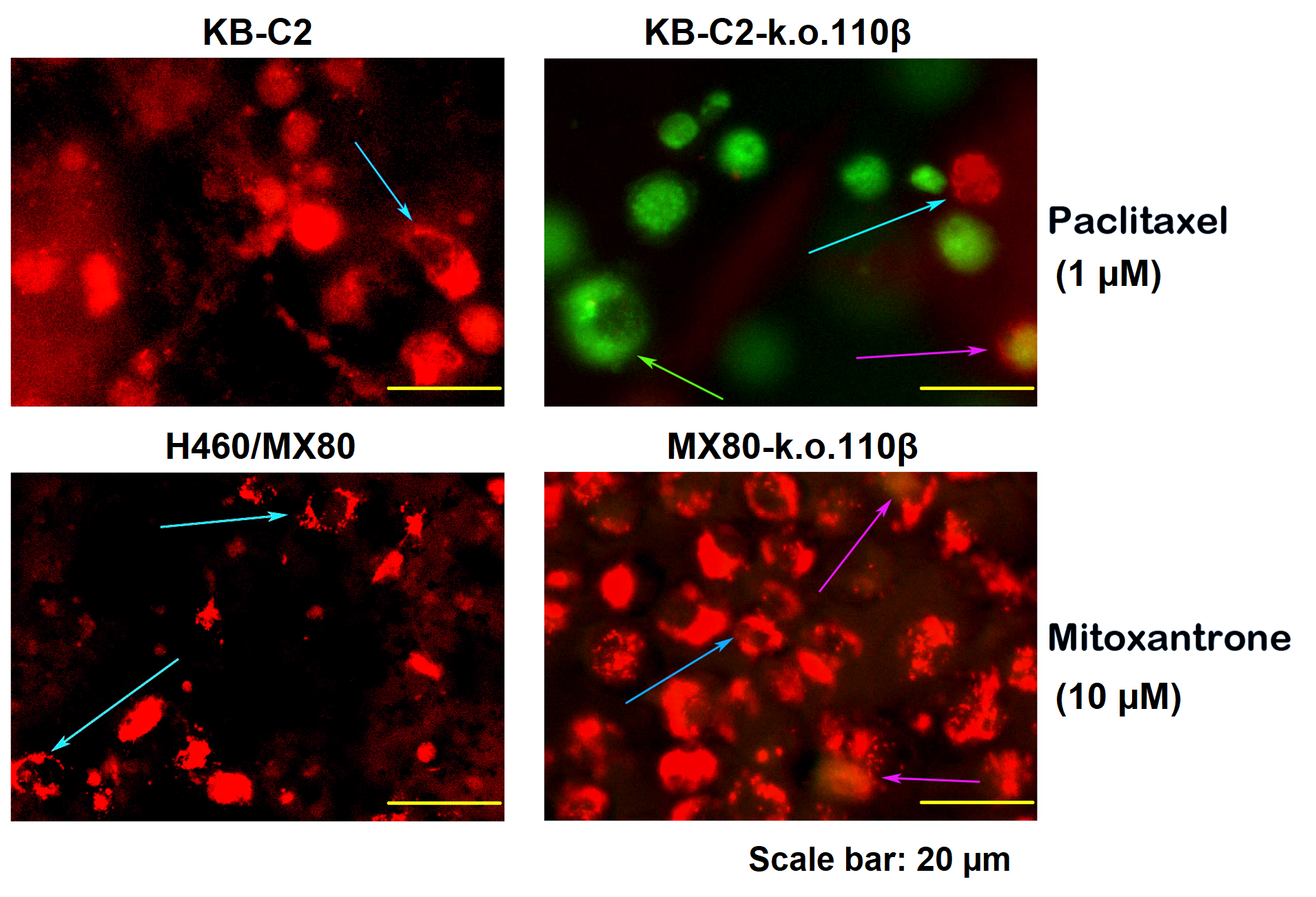


**Figure S5 JC-1 analysis of the apoptosis of KB-C2 and H460/MX80 cells and the derivative cells with deficiency of P110β.** JC-1 staining indicating mitochondrial membrane potential showed severe depolarization of the mitochondrial membrane as a result of severe apoptosis of 110β subunit-k.o. KB-C2 cells (namely KB-C2-k.o. 110 β), as depicted by the green arrows. KB-C2 and H460/MX80 cells without apoptosis are depicted by arrows in slight blue. Slightly apoptotic 110β subunit-k.o. H460/MX80 (namely MX80-k.o.110β) cells are depicted by arrows in pink. The KB-C2 and H460/MX80 cells were seeded into 96-well plates (5×10^3^ cells per well) and cultured for 8 h, followed by treatment with 1 µM of paclitaxel and 10 µM of mitoxantrone, respectively. JC-1 (2 µg mL^-1^) was then used for cell staining for 30 min. The cells were washed with PBS and observed with fluorescent microscope. The experiments were independently repeated three times.
